# Safety and preliminary efficacy of intranasal insulin for cognitive impairment in Parkinson disease and multiple system atrophy: A double-blinded placebo-controlled pilot study

**DOI:** 10.1371/journal.pone.0214364

**Published:** 2019-04-25

**Authors:** Peter Novak, Daniela Arantxa Pimentel Maldonado, Vera Novak

**Affiliations:** 1 Department of Neurology, Brigham and Women’s Hospital, Harvard Medical School, Boston, Massachusetts, United States of America; 2 Department of Neurology, University of Massachusetts, Worcester, Massachusetts, United States of America; 3 Department of Neurology, Beth Israel Deaconess Medical Center, Harvard Medical School, Boston, Massachusetts, United States of America; Emory University, UNITED STATES

## Abstract

Parkinson disease (PD) is associated with cognitive impairment. We aimed to determine the effects of intranasal insulin (INI) on cognition and motor performance in PD. This was a proof of concept, randomized, double-blinded, placebo-controlled trial evaluating the effects of 40 international units (IU) of insulin or saline once daily for four weeks on cognitive and functional performance. Of 16 subjects enrolled, eight in the INI group and six in the placebo group completed verbal fluency (FAS), Unified Parkinson Disease Scale (UPDRS), and modified Hoehn and Yahr scale (HY, PD severity) at baseline and post-treatment and were included in the analyses. After treatment, the INI group had a better total FAS score (p = 0.02) (41 ± 8.2 vs. 30.8 ± 7.1, mean ±SD, p = 0.02) compared to the placebo group. The INI group also had improved HY (p = 0.04) and UPDRS-Motor (Part III) (p = 0.02) scores when compared to baseline. One INI treated patient with multiple system atrophy (MSA) remained stable and did not show disease progression. The placebo group had no change. INI administration was well tolerated and there were no hypoglycemic episodes or serious study related adverse events or medications interactions. INI is safe in PD and MSA patients and may provide clinically relevant functional improvement. Larger studies are warranted to determine the INI effect in treatment of cognitive and motor impairment in Parkinson disease.

**Trial Registration:** ClinicalTrial.gov NCT02064166.

## Introduction

Parkinson disease (PD) is associated with a decline in cognitive performance and about 26% of patients diagnosed with PD develop mild cognitive impairment (MCI), more commonly the non-amnestic type [1–3]. The MCI incidence increases with age, disease severity and duration. MCI increases the risk for dementia and disability in PD patients [1,2,4] as well as the care-giver’s burden [5].

Cognitive impairment in PD has been associated with various mechanisms [6–9] such as regional cerebral perfusion deficit [10] and microstructural [11] and anatomical abnormalities. The microstructural abnormalities manifest even in PD patients with normal cognitive performance and become widespread as cognitive function deteriorates [6,11]. PD patients have altered functional connectivity within the resting state default mode network (DMN) [12] which regulates memory and other complex cognitive behaviors. The most affected pathways are connections between the posterior cingulate cortex, prefrontal cortex and medial temporal lobe [7,8,12] and long-range connectivity to other regions [13,14] that also demonstrate gray and white matter atrophy [13,14]. Walking speed is an important indicator of overall functional health and is correlated with survival in older adults [15]. Slower walking may be an early indicator of deterioration of attention and executive function underlying progression of cognitive decline [16].

Insulin plays a key role in glucose metabolism in the brain where it exerts important neuromodulatory, neurotrophic, and neuroprotective effects [17]. Intranasal insulin (INI) administration acutely increased resting-state functional connectivity between hippocampal and DMN regions in patients with type 2 diabetes without affecting serum glucose [18]. INI improved verbal and visuospatial memory in older diabetic and healthy adults, likely via regional vasodilatation in the anterior cerebral circulation [19]. In patients with MCI and Alzheimer disease, INI treatment improved visuospatial working memory and verbal working memory [20,21]. The rationale is that central insulin resistance and consequently impaired insulin signaling in the brain may be the common pathways for cognitive decline with aging, diabetes and Alzheimer’s disease. In the brain, insulin has vasodilatatory and neurotrophic effects, and therefore INI potential benefits are mediated by other mechanisms than by improving peripheral glycemic control. The effects of INI administration on cognitive performance in patients with PD are yet to be elucidated. We evaluated the cognitive and functional effects of the daily administration of 40 international units (IU) of INI in adults with PD over a four week period as compared to placebo (sterile saline) administration. We hypothesized that INI would improve verbal cognition and motor disability in non-demented PD participants after the four weeks of treatment when compared to placebo.

## Materials and methods

This was a pilot single-center, double-blinded, placebo-controlled study with parallel design thatevaluated the safety of INI on cognitive function in patients with PD. The study was conducted at the Autonomic Laboratory at the University of Massachusetts Medical School in Worcester, Massachusetts, United States. The study was registered at www.clinicaltrials.gov, number NCT02064166.

### Participants

Subjects were recruited from the Movement Disorders Clinic at the University of Massachusetts Memorial Medical Center. The Institutional Review Board at the University of Massachusetts approved the study. Participants were screened over the phone and eligible participants signed the informed consent form, as approved by the Institutional Review Board at the University of Massachusetts.

Inclusion criteria were: men and women older than 17 years with a clinical diagnosis of PD or MSA. Exclusion criteria were: pregnant or lactating women, patients with significant systemic illness that may interfere with the trial, a history of dementia, participants unable to walk for more than one minute, a history of allergic reaction to insulin, and nasal cavity inflammation that may prevent the absorption of insulin.

### Intervention and randomization

Participants were treated with 40 IU of human insulin (Novolin R Novo Nordisk, Bagsvaerd, Denmark) or placebo (sterile saline) delivered using the Via Nase device (Kurve technologies, Seattle, WA) once daily before breakfast for four weeks. The Via Nase device is an electronic atomizer that delivers the drug into the upper nasal cavity olfactory region, thus enhancing penetration into the brain.

The principal investigator (P.N.) who enrolled the participants, the study staff (D.A.P) who conducted the outcome assessment, and participants and their healthcare providers were blinded to the randomization assignment. V.N. was not involved in data collection. Subjects were randomized into the INI or placebo groups by the research pharmacy using the randomly permuted blocks method with two subjects per block (http://randomization.com). Insulin and placebo were distributed using the identical vials by the research pharmacy, thus blinding the participants and investigators.

### Protocol

Participants completed a screening visit, a baseline assessment, two follow-up visits, and an end-of treatment assessment over a four week treatment period. All participants completed detailed medical histories, neurologic physical exams and laboratory investigations (basic metabolic panel and pregnancy test in women of childbearing age). Functional assessments included neuropsychological testing, disease severity scales (to evaluate motor function and disease progression) and a walking test. Functional assessments at baseline and post-treatment were conducted while participants took their usual medications. The last INI/placebo dose administration was on the day of post treatment assessment.

### Neuropsychological, clinical and motor assessments

The Montreal Cognitive Assessment (MoCA) test was used to assess symptoms of cognitive impairment [22]. The verbal fluency FAS test was used to assess phonemic fluency and verbal memory [23]. For FAS, participants were asked to name words starting with letters F, A and S over a one-minute interval. The Beck Depression Inventory (BDI) is a 21-item scale that was used to evaluate depressive symptoms [24]. The clinical and motor assessments included the modified Hoehn and Yahr Scale (HY) to evaluate the severity of PD and treatment response [25] and the Unified Parkinson Disease Rating Scale (UPDRS, version modified by the Movement Disorders Society) to clinically assess PD effects on motor, cognitive, and other functions [26]. UPDRS, a widely-used outcome measure in clinical trials, is a sensitive indicator of motor progression and has satisfactory interrater reliability [27,28]. Motor score was calculated as proposed by Van Rooden et al. [29]. Bradykinesia score was calculated from the UPDRS item 23 + 24 + 25 +26 + 31 bilaterally. Motor asymmetry was estimated using lateralized UPDRS scores (item 20–26) (UPDRS I-III) as suggested by Jankovic et al. [26]. The UPDRS sub-scores summarize: UPDRS-I intellectual, mood and motivation impairment; UPDRS-II eating, activities of daily living, walking and balance; UPDRS-III speech, tremor; bradykinesia finger tapping, postural stability, and body bradykinesia and dyskinesia. Neuropsychological tests (MoCA, FAS, BDI) were administered by the research fellow trained in the study procedures (D.A.P.) and clinical tests (HY, UPDRS) were administered by the study clinician (P.N.).

### Gait test

All participants completed a standard four meter walking test [30] at their preferred walking speed to assess normal walking speed, number of steps and average stride length. The time used to complete a four meter walk was recorded. Average stride length was calculated by dividing the walked distance by the recorded number of steps.

### Statistical analysis

The analyses were performed using JMP 13 (SAS, Cary NC). Baseline demographic characteristics were compared between the groups using the Kolmogorov-Smirnov and Chi-squared tests. Primary endpoints were cognitive function measures (FAS) and secondary endpoints were MoCA, HY, UPDRS and BDI. The study outcomes (cognitive function measures FAS, MoCA), depression score (BDI), PD severity (HY scale and UPDRS-Motor), vital signs, and laboratory measures were compared between the placebo and insulin groups at baseline and at the end of treatment using repeated measures MANOVA adjusted for age. We have determined that data came from a normal distribution using the Shapiro-Wilk test, p>0.05. The treatment effect (baseline vs. post-treatment) was also compared within the INI and within the placebo group using a paired t-test. The effect size of the total FAS score was measured using Cohen’s test for post-treatment data. A level of p <0.05 was considered significant. The FAS was used as the primary cognitive outcome measure. Based on our current study, with 14 subjects total, we would have 79% power to detect a post-treatment difference of 10.2 points in the FAS total score between the insulin and placebo groups, standard deviation = 7.1, alpha = 0.05. Analyses were conducted by the original assigned group.

## Results

### Participant flow

**Fig 1:**
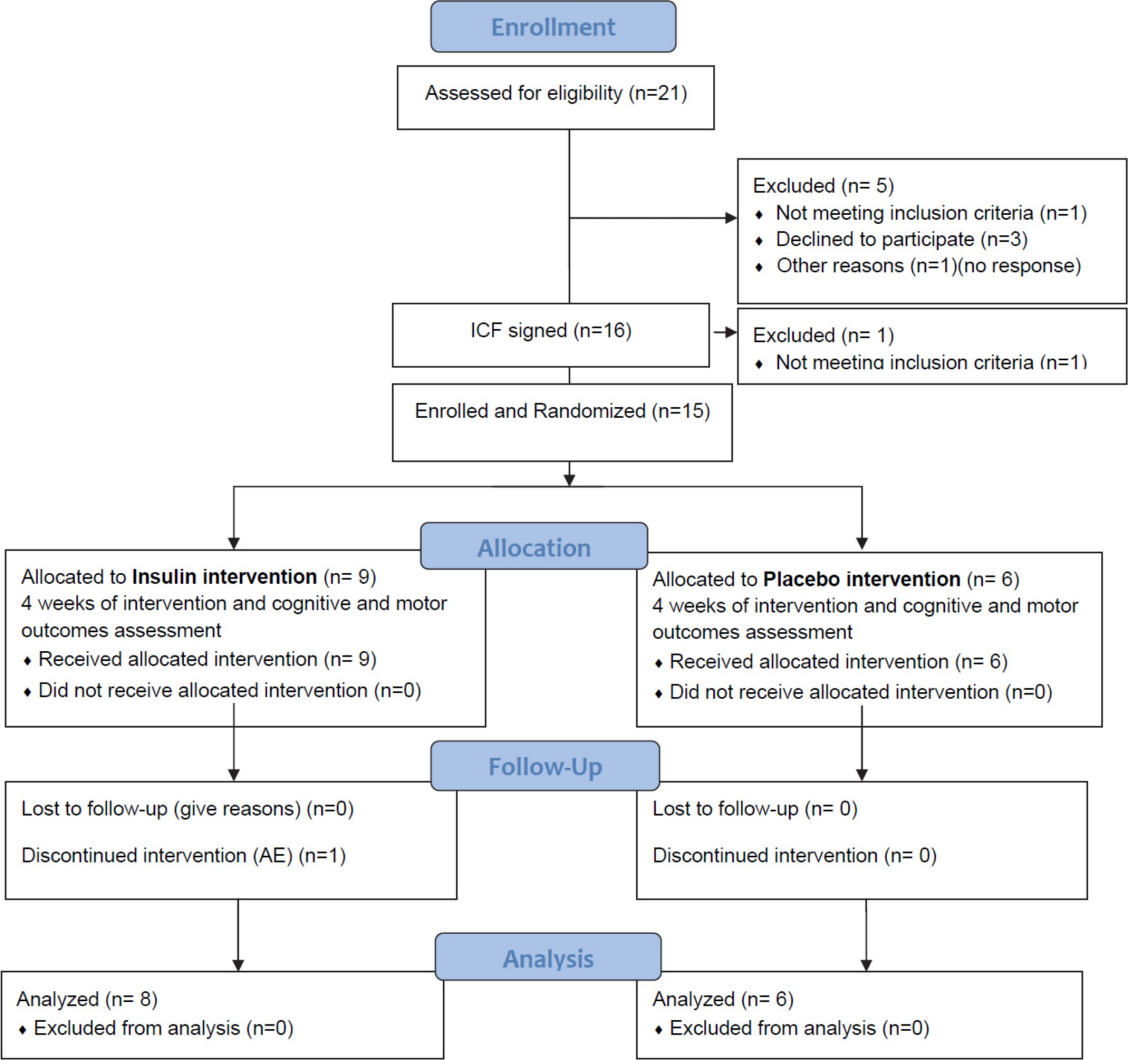
Study flow diagram.

Twenty-one patients were contacted over the phone to assess eligibility; five were excluded (one was not eligible, three declined to participate and one did not respond after initial contact) (Fig 1).

Sixteen subjects signed informed consents and one was excluded for dementia. Fifteen subjects were enrolled (eligible and randomized) with nine participants allocated to the INI intervention group and six to the placebo group. One subject from the INI intervention group was excluded for a study-unrelated adverse event. Fourteen subjects completed the study and were included in the analyses. Nine participants were treated with Levodopa (Sinemet or Sinemet ER), three with Stalevo-D, six with Pramipexol, one with Entacapone, nine with Rasagiline, two with Amantadine, and two with Ropinirole or combination of these medications. Participants were recruited between August 2014 and September 2015. The trial was stopped earlier than expected for administrative reasons.

### Neuropsychological, clinical, and motor assessments

The INI group included eight subjects (two women) and placebo group included six subjects (three women) (Table 1).

**Table 1:** Demographic characteristics of study population.

Group	Insulin	Placebo	p
N	8	6	
Age	63.3 ± 6.2	62.2 ± 9.6	0.59
Gender (M/F)	6/2	3/3	0.33
Race (W,AA)	7/1	6/0	0.27
Body Mass Index	23.9 ± 4.1	28.8 ±6.5	0.67
Disease duration (years)	6.8 ± 5.1	4.6 ± 1.7	0.72
Levodopa equivalent. (mcg)	733.8 ± 749.7	778.2 ±193.3	0.36

Mean ± SD, Kolmogorov-Smirnov and Chi-squared tests were used for between group comparisons.

Participants were diagnosed and treated for PD, with one subject in the INI group also treated for possible MSA. The INI and placebo groups had similar demographic characteristics, disease duration, and levodopa equivalent dose. At baseline, PD severity and neuropsychological test results (MoCA, HY scale, BDI depression, and FAS verbal fluency) were similar between the groups (Table 2).

**Table 2:** Effects of intranasal insulin/placebo treatment on verbal memory, motor function and vital signs.

Group	Insulin		Placebo		Between Groups	Within Insulin Group	Within Placebo Group
Treatment	Baseline	Post- treatment	Baseline	Post- treatment	Insulin vs. Placebo	Baseline vs. Post-treatment	
N	8	8	6	6			
Average stride (in)	22 ± 5.0	21.5 ± 4.4	21.0 ±2.7	19.2 ± 2.9	0.19	0.89	0.18
Walk duration (s)	3.8 ± 0.8	4.0 ± 1.0	4.4 ± 0.4	4.6 ± 0.7	0.20	0.57	0.16
Gait speed (m/s)	1.4 ± 0.3	1.1 ± 0.2	1.2±0.1	1.3 ± 0.3	0.12	0.61	0.21
No of steps	6.9 ± 1.5	7.0 ± 1.5	7.0 ± 0.9	7.7 ± 1.0	0.52	1.0	0.17
MoCA	28.7 ± 1.2	28 ± 1.1	26.8 ±2.6	28.2 ±0.9	0.15	0.17	0.31
HY	2.6 ± 0.6	2 ± 0.7*	2.4 ± 0.4	2.4 ±0.2	0.91	0.04	1.0
BDI	7.8 ± 5.4	8.25 ± 8.1	13.5± 5.6	12.8 ±7.4	0.20	0.75	0.72
**Verbal Fluency**							
F total	13.3 ± 2.7	13.6 ± 3.2	11.7 ±3.2	10.3 ±2.9	0.09	0.57	0.58
A total	11.4 ± 3.1	12 ± 3.2	9.8 ±1.9	8.5 ±2.9	0.08	0.59	0.16
S total	14.1 ± 3.0	15.4 ± 2.9	11.3 ± 2.2	12.0 ± 2.3	0.02	0.29	0.53
FAS total	38.8 ± 5.7	41 ± 8.2	32.8 ± 2.3	30.8 ± 7.1	0.02	0.37	0.49
**Unified Parkinson Disease Scale–Motor Score**							
UPDRS I	9.6 ± 3.9	8.12 ± 4.3	11.7±3.9	12.0±1.6	0.18	0.07	1.0
UPDRS II	13.9 ± 10.1	13.5 ± 10.8	15.3±8.2	14.0±8.2	0.89	0.64	0.18
UPDRS III	31.5 ± 20.0	25.6 ± 21.8*	31.7±13.3	30.5±15.8	0.84	0.02	0.27
Bradykinesia	13.1 ± 10.9	10.3 ± 10.9	14.42±8.2	12.8±7.7	0.76	0.08	0.82
**Laboratory values and vital signs**							
Fasting Plasma Glucose (mg/dl)	90.1±7.1	85.6±9.2	93.0±10.6	86.7±18.5	0.80	0.06	0.45
Heart rate (bpm)	68.6±6.7	73±9.8	74.2±14.6	86.3±16.0	0.51	0.58	0.24
SBP (mmHg)	117.7±9.4	123.8±10.3	130.8±14.9	117.3±17.0	0.75	0.35	0.23
DBP (mmHg)	71.6±7.1	76±8.9	79.2±6.8	73.3±7.1	0.74	0.24	0.22
Weight (lb)	170.3±40.5	169±38.4	187.3±62.9	186.7±62.8	0.66	0.39	0.42

Abbreviations: MoCA = the Montreal Cognitive Assessment; HY = Hoehn and Yahr Scale; BDI = Beck Depression Inventory; F,A,S = phonemic fluency and verbal memory; UPDRS = Unified Parkinson Disease Scale I-III; SBP = systolic blood pressure, DBP = diastolic blood pressure. Mean ±SD. MANOVA adjusted for age was used for between the insulin and placebo group comparisons at baseline and post-treatment. Paired t test was used within the insulin group and within the placebo group for comparisons between baseline and post treatment.

* Denotes differences between baseline and post-treatment <0.05 within insulin group.

### Verbal fluency–FAS

After four weeks of treatment, the FAS total number of words increased in the INI group at post treatment assessment, but decreased in the placebo group (p *=* 0.02) (lower-upper 95% 37.6–46.99) (Table 2). FAS increased in the INI group by 5.6% but decreased in the placebo group by 6.5%. The number of F words (p = 0.09) (lower-upper 95% 11.2–16.0) and A words (p = 0.08) (lower-upper 95% 9.7–14.4) tended to increase in both INI and placebo groups, however the number of S words (p = 0.02) (lower-upper 95% 13.3–17.4) increased only in the INI group. FAS scores were not different between baseline and post-treatment testing within the insulin group. FAS scores were not different between baseline and post-treatment testing within the placebo group. The effect size of the total FAS score between the INI and placebo groups was 1.33 on the post-treatment measurement. The total FAS score was within the age-adjusted normal range (38.5±13.7, mean ± SD) for 60–69 years old [31].

### Modified Hoehn and Yahr Scale–Severity of Parkinsonism

In the INI group, the modified HY scale score decreased after treatment when compared to the baseline (p = 0.04) (lower-upper 95% 1.6–2.43). This difference was not observed within the placebo group. HY score was not different between the groups, although it tended to worsen in the placebo group.

### UPDRS part III–Motor score

The UPDRS part III was lower after treatment in the INI group when compared to baseline (p = 0.02) (lower-upper 95% 9.74–41.5) (Table 2). This improvement in UPDRS part III was not observed in the placebo group. The UPDRS part I also showed a declining trend after treatment in the INI group when compared to baseline (p = 0.07) (lower-upper-lower 95% 4.99–11.3). The bradykinesia score also tended to decline in the INI group after treatment when compared to baseline (p = 0.08) (lower-upper 95% 1.96–18.5). There were no differences between the groups in UPDRS I = II and bradykinesia score.

### Cognitive and depression assessments

There were no differences between INI and placebo groups in MoCA and depression (BDI) at baseline and after treatment.

### Gait assessment

At baseline, gait speed, average stride length, and walk duration were within normal limits and similar between the INI and placebo groups. There were no significant changes in these parameters after treatment in either group.

### Multiple system atrophy case #1

One participant in the INI group (50 years old, F) was diagnosed with probable multiple system atrophy (MSA) two years prior to this study. The patient remained stable over the four weeks treatment period and did not show a significant disease progression. Baseline and post-treatment scores were similar with a trend toward improvement: MoCA 30 vs. 27; HY 2.5 vs. 2.5; FAS 44 vs. 46; BDI 11vs.12; UPDRS-I 9vs. 8; UPDRS-II 18 vs. 20; UPDRS—III 36 vs. 34; bradykinesia 20 vs. 18.

### Adverse events and safety

The INI treatment was well tolerated; there were no hypoglycemic episodes, nasal irritation or allergic reactions to insulin. One participant in the INI group developed pneumonia and thrombocytopenia that was unrelated to the study and was excluded. There were no study-related adverse events. There were no obvious interactions between the study drug and patient’s medications for PD.

### Vital signs and laboratory measures

There were no significant differences or trends in fasting serum glucose, heart rate, or systolic and diastolic blood pressure pre- and post-treatment in the INI and placebo groups (Table 2). There were no hypoglycemic episodes.

## Discussion

This prospective, placebo-controlled, double-blinded study compared the effects of the daily administration of 40 IU of Novolin R to sterile saline intranasally over a four-week period in PD patients on cognitive and motor performance. The administration of intranasal insulin was safe, as it has also been shown in the prior studies [18,19,21,32]. There were no significant changes in serum glucose, no hypoglycemic episodes and no serious study-related adverse events. Participants were able to adhere to the protocol and administer study medications.

At baseline, the INI and placebo groups had similar degrees of cognitive and motor performance that were within normal range for participants in the sixth/seventh decades [31]. Insulin administration over a four-week period improved the total FAS score, which is a measure of verbal fluency, when compared to the baseline and placebo group in the repeated measure design. The FAS score increased in the INI group by 5.6% but decreased in the placebo group by 6.4%. However, paired comparisons between baseline and post-treatment assessments were not significant in the insulin and in the placebo group due to a small sample size. Verbal fluency involves successful retrieval of information from memory. It requires attention and concentration, as well as the accomplishment of other executive cognitive tasks, which are the cognitive domains affected by PD. Further studies are needed to determine whether INI may provide benefits to PD patients even before they develop clinically significant cognitive impairment.

The treatment effects on motor performance and functionality were evidenced by the insulin group participants’ lower disability score on the HY scale as compared to the placebo group, which is a clinically significant finding. There was also an improvement in the UPDRS-Motor score (part III) in the insulin group when compared to baseline, but not in the placebo group. The placebo group showed a tendency toward decreased verbal fluency, HY score, and UPDRs score over a one month period.

One patient in the treatment group was also diagnosed with probable MSA, which is a rapidly progressing degenerative disease that has much shorter survival than PD [33]. The patient remained stable during the treatment period and did not show a progression of symptoms, but perhaps displayed a trend toward improvement on UPDRS scores. Although this is a single case of INI treatment in MSA, it warrants further investigation as there are no therapies available to modify disease progression.

Patients with PD are known to have a worse cognitive performance when compared to the general population. Mild cognitive impairment (MCI) is common in PD patients without dementia, affecting about 25% of patients. The cognitive domains affected are memory, visuospatial, and attention/executive function impairment, with memory most commonly affected (13% of patients) [4,13]. Non-amnestic MCI observed in patients with PD affects different cognitive domains, such as verbal memory, concentration and visuospatial memory [1]. Although our cohort was not diagnosed with PD-related MCI, they still demonstrated an improvement in verbal memory after the administration of INI over four weeks of treatment.

The cognitive decline observed in PD population has been attributed to impairment within the connectivity DMN as well as to impairment in regional perfusion [10]. PD patients with MCI show selective decline in interconnectivity in the non-motor networks, and specifically between the bilateral lentiform nuclei and superior parietal lobules and precuneus, primarily affecting the dorsal attention network [14] as well as the orbitofrontal cortex and other regions [7]. PD mainly affects long-range connectivity, as well as topological features of the brain networks related to memory, executive function, and visuospatial orientation [14].

Prior studies have demonstrated that INI administration acutely increased resting-state functional connectivity between hippocampal and DMN regions and improved verbal and visuospatial memory in type 2 diabetes and healthy adults [18]. This cognitive enhancement has also been attributed to vasodilatation in the middle cerebral artery [19], which perfuses cortical regions involved in language and attention performance.

This pilot study evaluated safety and feasibility of INI treatment in PD patients. The INI administration was safe; patients were able to follow the protocol and complete daily administration of INI without difficulties. However, the sample size of our study was small and therefore a larger study is needed to validate the results. In addition, our PD population was relatively well-functioning with only mild cognitive deficits. The study sample did not allow enrolling participants with more rapidly progressing MCI or dementia. Our battery of tests were selected to overlap with assessment of cognitive domains diagnostics for MCI (general cognition, verbal fluency and visuospatial orientation) [3] but also with tests that have successfully shown the effects of intranasal insulin therapy in other studies [19].

Therefore, in future studies we plan to include a larger patient population with more widespread motor and cognitive deficits. A longer duration of treatment may be needed to achieve long-term effects.

### Limitations

This was a pilot study and thus has several limitations, such as a small number of participants, relatively short duration of the study, and the administration of only one dose (40 IU) per day. There were also no participants with significant cognitive impairments, who may benefit the most from the insulin treatment. PD medications e.g. pramipexole, amantadine, and ropinirole may have adverse effects on mood and cognition, however they cannot be stopped for ethical reasons, and their doses were stable during treatment period. The sample size of this study was small and therefore, we could not conduct extensive statistical adjustments for medications, disease duration, glucose levels or other potential confounders. We can only presume that observed changes were the treatment effect rather than test–retest effect, because the placebo group has shown a trend toward lower cognitive and motor scores. Several studies have shown that INI is safe, is not associated with severe side-effects and does not affect glycemic control or causes hypoglycemic episodes [18,19,21,32]. Therefore, INI can be safely used in nondiabetic population, such as PD, without the risk of hypoglycemic episodes. Our PD patients had normal fasting serum glucose and did not have diagnosis of diabetes or prediabetes, and therefore hemoglobin A1c was not measured in this study. Insulin in the brain has vasodilatory and neurotrophic effects, and therefore potential benefits of INI action are mediated by other mechanisms than by improving peripheral glycemic control.

## Conclusions

This pilot longitudinal study has shown that INI administration may improve functional motor skills in PD and may potentially preserve cognitive performance as compared to baseline and the placebo group. This proof-of-concept approach has shown that the use of INI in treatment of cognitive and motor impairment related to PD is safe and feasible without the risk of hypoglycemia. The lack of disease progression in the MSA case warrants further investigation. Our study provided preliminary data that suggested an improvement of functional skills after four weeks of daily INI treatment that paves the way toward a larger cohort study to evaluate long-term safety and potential efficacy of intranasal insulin administration for potential treatment and prevention of functional decline in patients with Parkinson disease.

## Supporting information

S1 File CONSORT 2010 checklist: S1 CONSORT 2010 Checklist(20).doc. (DOC)

S2 File Study protocol: Protocol.docx. (DOCX)
