# Supplementary material for: Safety and preliminary efficacy of intranasal insulin for cognitive impairment in Parkinson disease and multiple system atrophy: A double-blinded placebo-controlled pilot study
Source: PLoS One. 2019 Apr 25;14(4):e0214364. doi: 10.1371/journal.pone.0214364 (PMC6483338; doi:10.1371/journal.pone.0214364)
Supplement: S2 File — (DOCX) [file pone.0214364.s002.docx]

**A Double-blinded Placebo-controlled Single-center Study to Evaluate the Efficacy of Intranasal Insulin 40 IU/day as Treatment for Subjects with Parkinson Disease and Multiple System Atrophy.**

**PROJECT SUMMARY**

Parkinson disease (PD) and multiple system atrophy (MSA) are progressive neurodegenerative disorders characterized by abnormal accumulation of α-synuclein (α-syn). There is no effective treatment t that can slow down the disease progression and both disorders are associated with severe cognitive decline. We showed that intranasal insulin (INI) treatment improves learning and memory in healthy and cognitively impaired non-diabetic adutls. Our proof-of-concept, randomized, placebo-controlled, cross-over pilot study (5R21-DK-084463-02, IND 107690, NCT01206322)^23^ has shown that a single 40 IU dose of intranasal insulin improved visuospatial memory in diabetes and control subjects. No serious adverse events or hypoglycemic episodes occurred. This proposal includes randomized, double blinded, placebo-controlled trial of INI (40 UI of intranasal insulin daily) in treatment of PD and MSA. Each arm will involve 11 patients with PD and MSA. Total duration of the study will be 2 years. The primary goal is to assess the efficacy of INI in treatment of cognitive abnormalities in both PD and MSA. The primary efficacy end point will be change of the cognitive scale ratings. This study as potential to improve our understanding how insulin modulate with cognitive brain ability. Results of this study may have a direct clinical impact. .

**HYPOTHESES AND AIMS**

Parkinson disease (PD) and multiple system atrophy (MSA) are progressive neurodegenerative disorders characterized by abnormal accumulation of α-synuclein (α-syn)^1,2^.  PD affects about 5 million persons while estimated prevalence of MSA is 3/100 000. Current treatment for PD and MSA is symptomatic only and does not alter the natural history of the disease. The feared complication associated with advanced disease is cognitive decline. Even for PD that has much longer survival than MSA, the presence of dementia for last years of disease is associated with substantial reduction of quality of live and loss of independence^1^.

Insulin plays an important role in brain functioning. Diabetes mellitus type 2 accelerates brain aging^3^ and increases the risk for dementia, Alzheimer’s disease^4-6^ and Parkinson disease^7-8^. Insulin is a key neurotrophic factor in the brain^9-12^ where it modulates cognition through regulation of energy metabolism, neurovascular coupling and neuroprotection.^13^

Intranasal insulin (INI) enters the brain through perivascular channels and binds to the receptors in the limbic system and memory networks including the hippocampus, hypothalamus, and insular cortex^14,12,15^ without systemic glycemic effects. INI treatment improves learning and memory in healthy and cognitively impaired non-diabetic adutls.^15-23^ Our proof-of-concept, randomized, placebo-controlled, cross-over pilot study (5R21-DK-084463-02, IND 107690, NCT01206322)^23^ has shown that a single 40 IU dose of intranasal insulin improved visuospatial memory in DM and control subjects. No serious adverse events or hypoglycemic episodes occurred.

**These results provide strong support for long-term INI-therapy as a strategy to preserve cognitive functions**. **We propose a single center open label trial on the long term effects of INI on cognition and memory in patients with Parkinson disease and multiple system atrophy.**

**Hypothesis**: Intranasal insulin improves cognitive functioning including attention, memory and executive function in patients with Parkinson disease and multiple system atrophy.

**Aim:** To determine whether intranasal insulin improves cognitive functioning in patients with Parkinson disease and multiple system atrophy.

**Significance and contributions to current knowledge**

Our goal is to use this proof-of-concept study to obtain evidence about the effects of intranasal insulin on cognitive functioning in adults with Parkinson disease and multiple system atrophy. These data will serve as basis to design a larger study that will prospectively validate this concept. Intranasal insulin administration may provide a novel therapeutic target for prevention and treatment of cognitive impairment in Parkinson disease and multiple system atrophy. If successful, this approach may have significant impact on clinical management of both disorders.

1. **BACKGROUND AND INNOVATION**

A group of progressive neurodegenerative disorders characterized by abnormal accumulation of α-synuclein (α-syn); also termed the α-synucleinopathies, includes Parkinson's disease (PD) and multiple system atrophy (MSA)^1,2^.  PD affects about 5 million persons while estimated prevalence of MSA is 3/100 000. Current treatment for PD and MSA is symptomatic only and does not alter the natural history of the disease. Both disorders are progressing irrespectively of treatment and average survival time for PD is above 20 years while for MSA is only 7 years. The feared complication associated with advanced disease is cognitive decline. Even for PD that has much longer survival than MSA, the presence of dementia for last years of disease is associated with substantial reduction of quality of live and loss of independence^1^.

- **Insulin – a key neuromodulator in the brain**

Insulin has emerged as a key neurotrophic factor in the central nervous system, and as a promising therapeutics for treatment of amnestic cognitive impairment and Alzheimer’s disease (AD). Insulin’s role in the brain is different from its actions in the periphery. Central insulin acts as an important neuromodulator in cognition ^22, 38^, energy homeostasis, food intake, sympathetic activity, neuron-astrocyte signaling, synapse formation and neuronal survival.^43, 44^ Furthermore, insulin has been shown to reinforce signaling in the brain–reward dopamine–mediated limbic system and modulate behavioral responses to natural food and other reward stimuli.^45-47^ Insulin receptors (IRs) are expressed in numerous brain regions, namely in olfactory bulb, hypothalamus, cerebral cortex, cerebellum and hippocampus.^48, 49,50^ More broadly, IR distribution overlaps with expression of downstream proteins and isoforms in insulin-related pathways.^51^ ^50^ Insulin also contributes to cortical blood flow regulation, as evidenced by IR presence within the neurovascular unit e.g. in neurons, astrocytes and capillaries,^48, 49, 49,52,^ ^53^ and the small vessels’ wall.^54^ We anticipate that cerebral insulin may directly modulate neuron-astrocyte signaling through neurovascular coupling and autonomic control of vascular tone and thus enable better regulation of local and regional perfusion ^55^ as well as neuronal activity in responses to various stimuli.^56,57,58,17,59^

Neuroprotective effects of insulin^60^ have been demonstrated *in vitro as* inhibition of neuronal apoptosis by activation of protein kinase B, and *in vivo* studies as a regulator of phosphorylation of tau, amyloid precursor protein, and clearance of β-amyloid.^61, 61-63^ Other neuro-protective effects of insulin also involve uptake of gamma butyric acid by astrocytes, protein synthesis, upregulation of glucose metabolism, blood flow and modulation of central autonomic serotoninergic and monoaminergic pathways.^56^ Animal and neuronal culture studies suggested that insulin may reduce central sympathetic activity, norepinephrine and increase acetylcholinesterase bindings.^49,64^ Insulin also converges with leptin signaling pathways in energy regulation of ATP-dependent potassium channels.^43^

However, central IRs are dependent upon insulin transport from the periphery through the blood-brain barrier (BBB),^65, 66^ via transporter-mediated and diffusion transport.^50^ Insulin transport is nearly saturated during euglycemia, which further supports insulin’s role as a regulator within central autonomic and endocrine systems, in contrast to its role in peripheral glucose metabolism. Aging, obesity, diabetes and AD alter insulin transport to the brain.^50, 67, 68^ Type 2 DM decreases insulin sensitivity in the brain, insulin transport through BBB, IRs receptor sensitivity and alters glucose metabolism.^38, 43, 44, 69^ Glucotoxicity and endothelial dysfunction associated with chronic hyperglycemia further affect perfusion, vasoreactivity and metabolism^70,71,72^ and thus contribute to neuronal loss.^73,74,2^ Therefore, inadequate insulin delivery to brain tissue, resulting from these complex interactions, may affect neuronal activity in multiple regions, but namely in the cognitive and brain-reward systems that have high demands on energy.^12^

- ***Intranasal insulin pharmacokinetics***

Intranasal insulin administration (INI) delivers insulin directly to the brain receptors ^13,11,14^, thus bypassing the BBB and avoiding systemic effects.^14,^ ^8^ Intranasal insulin increases rapidly in cerebrospinal fluid (CSF) and binds to IRs in the olfactory bulb, and along trigeminal and autonomic pathways in the frontal lobe, limbic system, hippocampus, hypothalamus and other areas.^13,11^ IRs can also be found in autonomic centers such as dorsal root ganglia, nigro-striatal neurons, hypothalamus and hippocampus.^54,13,75,11, 76^ The olfactory bulb has the highest IR concentration and the fastest transport rate of any brain region.^50^ Insulin enters cerebrospinal fluid along the olfactory neurons and perivascular channels through the cribriform plate .^77,13^ Insulin can also access other regions even hours after administration throughslower axonal transport in olfactory neurons. ^78^ Ten minutes after INI administration (dose 40IU), insulin levels began to rise and peaked at 30 to 45 minutes as compared to placebo (insulin 1091±219.8 vs. placebo 603.2 ±34.6 AUC (pmol/lxmin),p=0.02), which showed no change in serum levels (insulin 3410±276.8 vs. placebo 3410±106.1 AUC (pmol/lxmin), p=0.22). After that, CSF insulin began to decline, but still remained mildly elevated even 80 minutes post INI.^8^

INI administration is not effective to control hyperglycemia because it results only in about 1-2% bioavailability in the serum as compared to intravenous route,^79^ although special preparation and ultra-rapid administration may enhance its availability up to 10-15%.^80-82^

- ***INI administration is safe, with no significant effects on serum glucose or hypoglycemic episodes***

Early studies of insulin as an aerosol ^8, 79^ and recent studies on ultra-rapid-special preparation insulin (Nasulin)^82^, and high doses of insulin (120IU) with special lecithin preparation have shown transient decreases of plasma glucose or a mild reduction of postprandial glucose levels. ^83^ However, recent studies evaluating efficacy of INI to lower hyperglycemia in insulin–dependent diabetic. ^84, 85,86^ or type 2 DM subjects ^84, 87^ *have failed to demonstrate similar benefits on plasma glucose levels* ^88^. In healthy young subjects, there was a small (4%), acute decrease of plasma glucose within 30 minutes, which normalized by 60 minutes post-administration, partially due to absorption or inhalation of small amounts of insulin.^89^ The single dose of 40IU INI that we propose did not have significant effects on serum or subcutaneous glucose levels ^90,91^ acutely or chronically.^92^ The same insulin dose had no effects on heart rate or blood pressure, unlike a higher dose of 160IU INI which transiently increased blood pressure^90^, though without long-term effects.**^.^** ^90^

- ***Intranasal insulin improves cognitive function in clinical studies***

The insulin resistance syndrome, characterized by chronic peripheral insulin elevations, reduced insulin activity, and reduced brain insulin levels, is associated with age-related memory impairment and Alzheimer’s disease (AD). ^93, 94^ These mild forms of insulin resistance may precede AD pathology for years.^38, 39^ The risk of type 2 DM for dementia and AD in late life has been increasingly recognized in population studies,^1,25,26^ and impaired insulin signaling in the hippocampus and hypothalamus, as seen in both conditions, may provide a common link between DM and AD^95^. The evidence that INI could be a promising treatment for improving cognitive function is growing. ^36, 38, 38, 96-102^ Clinical studies suggest that augmenting cerebral insulin improved performance in specific cognitive domains and memory in healthy young ^90,103,18^ and older adults^17,36,104^ and patients with mild cognitive impairment and AD ^17,36^ with both acute and chronic administration. In healthy men, INI also improved mood and regulated food intake^14,91.^

In healthy adults, INI administration of rapid-acting insulin (40 IU q.i.d.) for 8 weeks improved long-term declarative memory over regular insulin, though both types of insulin were better than placebo. No systemic side effects were observed, and serum glucose and insulin levels did not change ^34, 35^. In another study, patients with amnestic mild cognitive impairment (MCI) and mild-moderate AD were treated with 40IU (Novolin^®^ Novonordisk) for 3 weeks. The INI-treated group retained more verbal information, showed improved attention compare with placebo treated group. Acute INI administration facilitated verbal memory in memory impaired ApoE4- adults, with the best performance obtained with 20IU INI, though there was no improvement at 60IU INI. Additionally, the first clinical trial in 104 patients with amnestic MCI or with mild-moderate AD over a 4 months period has shown that 20IU INI (10 IU b.i.d.)(Novolin^®^) improved delayed memory, and both 20IU and 40IU (20 IU b.i.d.) doses preserved caregiver-rated functional ability and general cognitive function (REFS?). Cognitive performance was better with 20IU (10IU b.i.d.) dose in this population as compared to the 40IU dose^21^. These findings are highly relevant to our proposal because of the high prevalence of dementia in DM patients (Rotterdam study) as well as the high prevalence of insulin resistance in AD patients.^94, 105^ ^38, 106^ Furthermore, our preliminary results also showed that improved performance on visuospatial and verbal tasks in older adults and type 2 DM patients correlated with vasodilation in the middle cerebral artery (MCA) territory. Functional MRI studies that showed increased activity in the brain–reward dopamine–mediated limbic system further support these findings. ^45-47^ Taken altogether, these data suggest that intranasal administration of insulin is a safe and feasible approach to improving central nervous system insulin levels. In addition, INI could be a promising treatment for disorders that involve disturbances in brain insulin signaling, such as AD, obesity, and type 2 DM.^107^

- ***INI increases resting blood flow, energy in the brain and signaling and brain-reward system***

An acute INI administration of 40IU increased resting blood flow in insular cortex as compared to placebo and cortisol in healthy young men.^41^ Voxel-based analyses identified other clusters with correlations between insular cortex activity and the opercular part of the inferior frontal gyrus, putamen and left caudate activities. Insular cortex is a primary central autonomic and gustatory site (REFS?). No rCBF changes were observed in hippocampus or in visual cortex.^41^ The activation of the right insular cortex has been associated with improvement in cognitive tasks that are challenging and require longer processing as well as simpler tasks (SUCH AS?) in older or impaired individuals,^108^ in addition to being linked with autonomic system arousal.^109^ Furthermore, Jau-Chara et al. demonstrated with 31pMR spectroscopy that the same dose of 40IU INI acutely increases brain energy metabolism in the motor cortex that persisted over 100 min after administration.^110^ Increased neuronal activity was also observed in the brain–reward system, which includes hypothalamus, right insular cortex, orbitofrontal cortex and putamen observed after 40IU INI dose, that persisted for one hour after administration.^111^ Over a 4 month period the AD and MCI groups treated with 40IU (20IU b.i.d.) showed less progression of hypometabolism in bilateral frontal, right temporal, bilateral occipital, andright precuneus and/or cuneus regions, while placebo group showed reduced neuronal activity in these same regions^21^.

- ***Cognitive response to insulin may be dependent upon vasoreactivity***

Hyperglycemia-induced small vessel disease is a common pathway for abnormal blood brain barrier (BBB) function, neurovascular coupling, regional vasoreactivity and hypoperfusion, neurotoxicity and neuron-astrocyte signaling in type ^22,3,4,71^.

Small vessel disease presents as lower baseline perfusion, blunted vasodilatation to hypercapnia and exaggerated vasoconstriction to hypocapnia affecting multiple vascular territories across several anatomical regions e.g. frontal, parietal and occipital lobes. ^2,27^ Cerebral perfusion and vasoreactivity negatively correlate with the degree of insulin resistance, glycemic control and vascular inflammation.^7,2,27^ Vasodilatation-associated increases in blood flow via insulin-stimulated production of nitric-oxide ^58^ in vascular endothelium have not been well studied in the human brain. Therefore, vasodilatation to hypercapnia, although not a specific measure of endothelial function, may serve as an effective proxy to neurovascular coupling within specific regions, as well as the ability to redistribute blood flow to those regions ^112,27^. Therefore, INI may have direct effects on neurovascular coupling, regional vascular tone and neuronal activity.^57,58,17,59^ Decreased vasodilatation and increased vasoconstriction reactivity associated with DM are associated with regional gray matter atrophy and worse functionality in older diabetic adults.^27^ Conversely, the relationship between improved vasodilatation on insulin with improved cognitive scores may suggest vasoreactivity as a potential diagnostic tool for determining responsiveness to intranasal insulin therapy.^23^

The activation of the right insular cortex has been associated with better responses to cognitive tasks that are challenging, require longer processing or even to simple tasks in older or impaired individuals,^108^ or are linked with autonomic system arousal.^109^ MCA territory includes cortical areas representing learning and memory, as well as the insular cortex, which is an important relay region for autonomic functions, emotions and memory. In particular, right insular cortex provides a link across systems that are selectively responsive to attention-related problem solving during conditions that require attention and coordination during a task performance.^113^

- ***Sex-dependency of anorexogenic and peripheral insulin sensitivity effects***

Potential anorexogenic effect of INI would be of great interest for treatment of overweight type 2 DM patients. In animals, direct intraventricular insulin administration inhibits food intake, suppresses appetite, decreases body weight and may facilitate gluconeogenesis and increase serum glucose levels.^46, 114^ Recent functional MRI data has linked INI insulin to brain–reward limbic dopamine–mediated system which plays role in reinforcing behavioral responses to natural food and other reward stimuli.^45-47^ Increased activity in hypothalamus, right insular cortex and orbitofrontal cortex ( a part of pre-frontal cortex)and putamen observed one hour after administration correlated with lower HOMA-IR (increased insulin sensitivity)^89^. INI administration (40IU, single dose) reduced appetite and caloric intake ^110^, whereas a higher dose of 160IU (o.d.) resulted in gender-specific anorexogenic (men) and hippocampal-dependent memory improving effects (women).^115^ The INI-treated lean men lost 1.28 kg body weight on average, decreased waist circumference, and reduced plasma leptin by 27%.^116, 117^ However, while obese men, who may be insulin resistant, did not lose weight, they showed improved declarative memory. ^118^

- ***Long-term benefits in murine model of diabetic encephalopathy***

Francis et. al. ^64^ that reported the long term effects of INI administration in a streptozotocin-induced mice model over 8 months period on brain volumes and functional outcomes. In these mice, INI slowed development of cognitive decline during weekly cognitive/behavioral testing, ameliorated monthly MR imaging abnormalities, prevented quantitative morphological abnormalities in cerebrum, decreased mortality and reversed diabetes-mediated declines in mRNA for phosphoinositide 3-kinase (PI3K)/Akt and for protein levels of the transcription factors cyclic AMP response element binding protein (CREB) and glycogen synthase kinase 3b (GSK-3b) within different cerebral regions.

Radio-labeled insulin delivery revealed that intranasal administration delivered more rapid and substantial insulin increases within the cerebrum with less systemic insulin detection when compared with subcutaneous insulin delivery. Brain atrophy and white matter hyperintensities, common markers of diabetic changes in the brain, were significantly reduced. Diabetes-mediated losses of protein and mRNA for the synaptic elements synaptophysin and choline acetyltransferase were prevented with intranasal insulin delivery.

Recently , Yang et al^119^ replicated the findings in a type 2 DM murine model ,and showed that after 4 weeks of INI treatment AKT and GSK-3β normalized, and tau hyperphosphorylation in T2D rat brains was reduced, whereas four-week treatments with ***subcutaneous insulin*** had minimal effects on brain GSK-3β and tau phosphorylation.

In summary, there is growing evidence that INI could be a promising method for the treatment of disorders that involve etiology of central insulin resistance and involve abnormal insulin signaling within brain such as PD and MSA.

There is sufficient evidence that INI administration of 40UI of insulin (o.d.) is safe and feasible in older and cognitively impaired populations and that this INI dose is highly unlikely to significantly affect glycemic levels or severe hypoglycemic episodes.

Building upon promising preliminary results and other past studies in the literature, this study will provide further evidence about short-term and long-term effects of INI on specific cognitive domains and memory functions.

**C. METHODS**

This is a single center, double-blinded, placebo-controlled study. The study is designed to evaluate efficacy of intranasal insulin in Parkinson disease and multiple system atrophy. The project includes 4 visits, the screening visit, 2 treatment visits and one follow up visit. See the table I for a timing diagram. The total duration of the study is 2 years. The study will be conducted in University of Massachusetts.

**C.3.1. SUBJECTS:**

We plan to enroll 22 subjects with PD and 22 subjects with MSA to the trial, total 44 subjects. Every effort will be made to ensure that the study population reflects the makeup of general population including minorities.

**Patient selection**

MSA patients and healthy controls will be enrolled following NIH guidelines. Details of human subjects are **outlined in the Human** Subjects section.

**Inclusion criteria:**

1. Male or female older than 17 years.

2. Clinical diagnosis of Parkinson disease or multiple system atrophy^1,2^

3. Provide written informed consent to participate in the study

4. Understand that they may withdraw their consent at any time

**Exclusion criteria:**

1.Women who are pregnant or lactating

2.In the investigator’s opinion, have significant systemic, hepatic, cardiovascular, renal or other illness that can interfere based on investigator judgment with the trial.

3.History of dementia

4.Unable to walk without help for at least 1 minute

5.History of allergic reaction to insulin

6.The presence of inflammation of nasal cavity that may prevents absorption of insulin

**Standard clinical care:** For the duration of the study, participants will be under the care of their usual providers. Participation in this trial will not interfere with the patients’ standard care or any additional care that may be required while they are participating in the study. We will communicate with the patients’ physicians, as needed, regarding any new findings or issues that arise during the course of the research.

**C.4. PROTOCOL AND PROCEDURES**

The synopsis of the protocol and procedures is at Table 1.

1. **Screening visit.**

The eligibility of subjects to participate in the study will be evaluated in the baseline screening visit. After obtaining Informed consent form, enrolled subjects will be undergo evaluations that include medical history, physical exam, vital signs, gait analysis, scale testing as outlined in Table I.

1. **Treatment visits**

There will be 2 treatment visits (table I).

At the treatment visit the following will be obtained:

- Vital sings
- Scale testing
- Gait analysis
- Adverse events
- Intervention education
- Intervention

**Scale Testing:** These scales include:

- Unified Parkinson’s Disease Rating scale (UPDRS Parts I, II, and III) - the most commonly used rating scale in Parkinson’s disease for assessing symptoms,
- Patient Global Impression - Improvement scale (PGI-I), - a scale to assess your impression of whether your symptoms have changed
- Modified Hoehn and Yahr Scale - a scale to measure severity of Parkinson’s disease

| Table 1. Synopsis for the subjects. | | | | |
| --- | --- | --- | --- | --- |
| *Visits* | Screening | Treatment 1 | Treatment 2 | Follow-up |
|  | Visit 1 | Visit 2 (within a week after visit 1) | Visit 3 (2 weeks after visit 2) | Visit 4 (2 weeks after visit 2) |
| Inform. Consent & Eligibility | X |  |  |  |
| Medical History, Demography | X |  |  |  |
| Physical Exam | x | x |  | X |
| Vital Signs (BP, HR & weight) | x | x |  | x |
| Montreal Cognitive Assessment | x | x |  | X |
| Scales* |  | x |  | x |
| Walk analysis | x | x |  | x |
| Intranasal treatment (insulin or placebo) |  | x | X |  |
| Adverse events | x | x | X | x |
| Concomitant Medications | x | x | X | x |
| ^*^ Unified Parkinson’s Disease Rating scale (UPDRS Parts I, II, and III), Patient Global Impression - Improvement scale (PGI-I), Modified Hoehn and Yahr Scale, Beck Depression Inventory Score (BDI), Montreal Cognitive Assessment (MoCA), BVMT-R (Brief Visuospatial Memory Test-Revised), Verbal Fluency FAS test | | | | |

- Beck Depression Inventory Score (BDI) - a scale to measure levels of depression
- Montreal Cognitive Assessment (MoCA) - a scale to assess symptoms of cognitive impairment
- BVMT-R (Brief Visuospatial Memory Test-Revised- a scale to assess memory impairment
- Verbal Fluency FAS test – scale to test verbal fluency

**Gait analysis**

*W*alking speed reflects health and functional status and has been recommended as a potentially useful clinical indicator of well-being among the older adults^202^. Standard 4-meter test will be used^202^. The following parameters will be obtained: walking speed, number of steps and average stride length.

**Intervention**

We will use Novolin R^203^, a commercial preparation that routinely used for the treatment of diabetic patients.

**Preparation and administration of the insulin**

Novolin R 100 units/ml that is routinely used in diabetic patients. The insulin will be administered using Vianase, an electronic atomizer (Kurvy Technology) that is a nasal drug delivery device (<http://www.kurvetech.com>). The device is simple to use and was designed for self-administration. The device uses the standard 100 U /ml vials that are simply inserted into the device.

Rationale for the dose:

The dose used in this trial was used in previous studies^23^.

Randomization

Half of the study subjects (50%) will receive insulin and half placebo. The randomization will be done by a research pharmacy. Normal saline has similar consistency and transparency as insulin and it will be used as a placebo. Research pharmacy uses standardized protocol described at [http://randomization.com](http://randomization.com/) to create a random list based on number of subjects and block size requested. Research pharmacy will keep the list in the IDS pharmacy study binder. When a subject is enrolled the pharmacist will record the subject name in the next available entry on the list to determine treatment group. The list is for pharmacy use only and not available to the PI of other study support staff. Both placebo and intranasal insulin will be distributed at equal vials that will be compatible with the Vianase device.

Criteria for early termination – removal from the study

In the absence of a medical contraindication or significant protocol violation, every effort will be made by investigators to keep the subject in the study. However, should the subject decide to discontinue treatment, all efforts will be made to arrange a final evaluation with an explanation of why the subject is withdrawing from the study. All subjects who prematurely discontinue from the study will receive a follow-up telephone call and the final follow-up visit will be arranged (refer to Section Final follow up visits/termination visit).

The study treatment will be stopped if any of the following events occur:

• The subject has any AE, laboratory abnormality, intercurrent illness, or other

medical condition that may requires the subject to stop taking insulin.

• The subject decided to withdraw consent from the study;

• The Investigator concludes that it is in the best interest of the subject to discontinue study

treatment;

• Subject is noncompliant;

• If pregnancy is suspected while the subject is receiving study treatment, the study

medication will be immediately stopped until the result of pregnancy testing is known.

If pregnancy is confirmed, the study medication will be permanently discontinued and the

subject withdrawn from the study. Reasonable attempts will be made to follow the pregnancy to conclusion in order to obtain information regarding the outcome.

1. **Final follow up visits/termination visit**

The follow up visit will be scheduled about 2 weeks after the second treatment visit. Subjects who prematurely dropped out of the study will be contacted by telephone for a follow-up visit within 7 days after their last dose of study drug.The follow up visit include scale testing, gati analysis, adverse events, concomitant medication and vital signs (table I).

**C.5. DATA ANALYSIS, STATISTICAL ANALYSIS, AND RESULTS INTERPRETATION**

**Statistical Analysis and Expected Results**

**Sample Size Power Estimation**

This is a pilot study as the intranasal insulin was not used in PD or MSA patients. To estimate the power for this study for the primary outcomes, MOC, we can use the results from the previous pilot study^23^ using other patients to estimate the change in the insulin--treated patients and, from the literature, the change in the placebo patients.

For BVMT we estimate an improvement of 7.0 points during the trial compared to baseline and a standard deviation of 6.0. For the sample size 20 for each diagnosis (10 subjects in each arm, e.g. will have 20 patients with PD form them 10 treated with intranasal insulin and 10 with placebo, and will have 20 patients with MSA form them 10 treated with intranasal insulin and 10 with placebo) using an unadjusted two-group t-test with equal variances and a one-sided alpha-level =0.05, we will have over 80% power to detect a difference of 7 points between the baseline and after the treatment scores (less than hypothesized above).

We expected drop out 10% and the enrollment size was adjusted accordingly.

**Analysis section**

**Endpoints:**

Based on our previous study^23^, the changes in the score of the BVMT-R (Brief Visuospatial Memory Test-Revised will be the primary efficacy endpoints.

The secondary efficacy endpoints will be the Montreal Cognitive Assessment l Unified Parkinson’s Disease Rating scale, Patient Global Impression, Modified Hoehn and Yahr Scale, Beck Depression Inventory Score.

**Statistical analysis:**

Our initial statistical procedures will be descriptive in nature, concentrating on means and standard deviations as well as medians and interquartile ranges (IQR).

Our statistical comparisons will test the difference in the continuous outcomes of the scale scores using standard ANOVA tests and, for sensitivity, a non-parametric test (e.g., Mann-Whitney/Wilcoxon test for central tendency). We will use mixed effects models to model the individual patient trajectories over the course of treatment with the same outcomes (scale scores) and, as predictors, treatment and time (treatment-by-time interaction). The individual patient trajectories will allow us to identify any patients who did particularly well (or not very well) to determine any factors that might allow us to tailor the treatment to particularly responsive patients in future studies.

For any categorical outcomes, such as occurrence of AEs, SAEs, or safety measures, we will use contingency table analysis with Fisher’s Exact Tests as the statistical test.

**Expected results:** We expect that intervention with nasal insulin will improve the BVMT-R (Brief Visuospatial Memory Test-Revised and other cognitive testing scores.

**C.6.1. PRELIMINARY RESULTS**

Our preliminary results were published recently^23^. We designed the study to determine acute effects of intranasal insulin on regional cerebral perfusion and cognition in older adults with type 2 diabetes (DM). This was a proof-of-concept, randomized, double-blind, placebo-controlled intervention evaluating the Q:2 effects of a single 40-IU dose of insulin or saline on vasoreactivity and cognition in 15 DM and 14 control subjects. Measurements included regional perfusion, vasodilatation to hypercapnia with 3 Tesla MRI, and neuropsychological evaluation.Intranasal insulin administration was well tolerated and did not affect systemic glucose levels. No serious adverse events were reported. Across all subjects, intranasal insulin improved visuospatial memory (P< 0.05). In the DM group, an increase of perfusion after insulin administration was greater in the insular cortex compared with the control group (P = 0.0003). Cognitive performance after insulin administration was related to regional vasoreactivity. Improvements of visuospatial memory after insulin administration in the DM group (R2 adjusted = 0.44, P = 0.0098) and in the verbal fluency test in the control group (R2 adjusted = 0.64, P = .0087) were correlated with vasodilatation in the middle cerebral artery territory. We concluded that Intranasal insulin administration appears safe, does not affect systemic glucose control, and may provide acute improvements of cognitive function in patients with type 2 DM, potentially through vasoreactivity mechanisms. Intranasal insulin-induced changes in cognitive function may be related to vasodilatation in the anterior brain regions, such as insular cortex that regulates attention-related task performance.

**C.7. POTENTIAL PITFALLS AND LIMITATIONS**

Several potential pitfalls can arise during the study.

1.Severe adverse events. Even previous studies showed that intranasal insulin is safe, severe AE can happen and can influence the outcome of the study. This scenario will be reduced by careful education of patients regarding the side effects and careful selection of the study subjects.

2) Subjects unable to complete the study because of PD or MSA progression. We plan to enroll both disorders at earlier stages thus increase probability that subjects will be able to complete the study.

**E. HUMAN SUBJECTS**

**Protection of Human Subjects**

The proposed study will be conducted on human subjects. UMMS Institutional Review Board will review the study protocol. Informed consent will be obtained from all patients enrolled in this trial. Subjects will be informed of their right to withdraw from the study at any time. Relevant issues to the protection of subjects are outlined below.

**Risks to Human Subjects**

**Subject recruitment**

The characteristics of subjects, inclusion and exclusion criteria are described in the Methods section Table. Study subjects will be recruited from the Department of Neurology, University of Massachusetts. Interested subjects will be contacted by a study coordinator. Interested subjects will be ask to tome to the Department of Neurology clinic for a screening visit. Subjects will be provided with the consent form for their review prior to the screening visit. During the screening visit, the study investigator will obtain informed consent after details of the study have been presented. All subjects will have an opportunity to ask questions. The study subjects will sign the consent form if he agree and he will receive a copy of the signed consent form*.*  All collaborating investigators have extensive experience in conducting clinical trials, including and ability to enroll subjects. The investigators have also extensive experience with the follow-up studies and all techniques proposed in the current trial.

**Sources of research material**

Participating human subjects will be the source of clinical data. Confidentiality and data safety measures will follow HIPAA regulations implemented at the University of Massachusetts Medical School. Research data on human subjects will be obtained from medical records, patient interviews, physical examinations, routine clinical blood tests, electrocardiogram recordings, Doppler ultrasound images, MRI, and specific methods used in the study protocol such as scales and gait results as described in the study Protocol Section. To maintain confidentiality, each subject enrolled in the study will be assigned a unique number and entered into the electronic database. Files will be stored in a password-protected computer network and locked cabinets. Subjects may authorize us to inform their primary care physician of results relevant to their care. Identifying information about subjects will not be used in the discussion, presentation, or publication of any research data.

**Risks and discomforts**

The pharmacological intervention and all testing procedures within the proposed study will utilize well-established physiologic monitoring and are associated with minimal risks.

Intranasal insulin was used extensively in many studies and is considered to be safe.

Several hundred people have received doses of intranasal insulin in previous clinical studies most of these being patients with Alzheimer disease. Most of the adverse events were minor. The most common risks seen in previous clinical studies of intranasal insulin are:

- Light-headedness and/or dizziness
- Headache
- Nose bleed
- Rhinitis
- Upper respiratory tract infection
- Falls
- Rash

For any new medication it is possible that there are side effects that have not been noted in previous clinical studies.

Rating scales: Minimal risk is associated with performing the rating scales. Subjects may become mentally stressed or exhausted. Rest will be given if necessary, and testing will be stopped upon subject request.

Walking test: Walking test at preferred speed with and without cognitive challenges is associated no more than minimal risks. Physical activity associated with the test is low to moderate intensity, and potential risks include strains, sprains, muscle soreness, and light-headedness. In rare instances, more serious side effects such as an injurious fall may occur.

There is additional risk of losing confidentiality.

**Adequacy of protection against risks**

**Informed consent**

In accordance with US FDA regulations (21 CFR 50) and ICH-GCP Consolidated Guidelines (Federal Register, May 9, 1997, Vol. 62, Number 90), informed consent will be required from all subjects, or their legally authorized representative, prior to their participation in this study. At the initial contact with a potential candidate, the investigator(s) will provide an adequate explanation of the purpose, procedures, possible risks/benefits, and participant responsibilities, in addition to the fact that his/her participation is voluntary, that he/she may withdraw from the study at any time, and that the decision not to participate or to withdraw will not affect subject’s care in any way. Potential participants will be given ample opportunity to ask questions and to consider their decision. If the subject expresses a sustained interest, a written informed consent will be obtained before any study-related test/procedure is undertaken. A copy of the consent form will be given to the participant. Patients with a known history of dementia will be excluded, thereby minimizing the possibility of invalid informed consent.

**Procedures for protection of subjects**

Safety monitoring procedures will be implemented and reviewed by a Safety Monitoring Board, in accordance with NIH safety policies for human intervention studies (NIH Guide, 25 (33), October 4, 1996). At each encounter between the subject and the researchers in person or by telephone during the study, MRI and physiological studies, standardized adverse event monitoring forms will be completed. All of these reports will be summarized into tables and to members of the Safety Monitoring Board.

**Safety monitoring plan**

The Principal Investigator and co-investigators have extensive experience with safety procedures and the following safeguards will be instituted to further minimize the possibility of adverse events. Study investigators (Dr. P. Novak and Dr. Ravin) will oversee subjects’ safety and will be available for medical emergencies. There is coverage for investigators at Department of neurology UMASS provides coverage for the study investigators continuously for 24 hours daily.

**Reporting of adverse events.**

The study will comply with Food and Drug Administration (FDA) regulations requiring reporting of adverse events (AE) associated with the use of a study drug. We will use NIH criteria for definitions of AEs. Serious adverse events that are unexpected and associated with the study drug will be reported within 24 hours to the IRB. All AE’s will be reported to IRB.Adverse side effects will be monitored and recorded during each visit.

**Data Safety Monitoring Board.**

We will establish a Safety Monitoring Board (SMB). The SMB members will be experienced clinical investigators that are not co-investigators of this study except of PI. The SMB members will be recruited from the faculty members of Department of Neurology at UMASS. The SMB members will review the study protocol and safety monitoring data collection forms, proposing additions or changes needed for review and assurance of subject safety. The Board will then meet twice a year to review standardized reports of subject symptoms, deviations from the study protocol, and treatment decisions. They will review the progress of recruitment and retention of subjects, compliance with the protocol, and operating procedures. If they raise concerns about safety issues, they may request additional data and propose specific analyses. They will make recommendations to the Principal Investigator regarding recruitment, retention, compliance, and safety issues. Before each meeting, the Board will be sent reports of research activity and summaries of safety monitoring information. The Investigators agree to cooperate with the SMB to ensure that any problems detected in the course of these monitoring visits are resolved.

**Risks in relation to anticipated benefits**

Based on previous research with this and similar protocols, we judge the risks associated with this study to be minimal as intranasal insulin was shown to be safe in many previous studies. Individual subjects may benefit from the proposed study by learning more about the PD and MSA.

**Payments**

There will be no payment for participation in the study.

**Importance of the knowledge to be gained**

The findings from these studies are expected to provide important information regarding the effects of intranasal insulin in PD and MSA. These results may ultimately lead to multi-center trials and modification of treatment guidelines for both disorders.

**References**

1. Olanow CW and Schapira AH. Therapeutic prospects for Parkinson disease. Ann Neurol 2013;74:337

2. Ubhi KU, Low P, Masliah E. Multiple system atrophy: a clinical and neuropathological perspective.

Trends in Neurosciences 2011, 34:581-590

3. Xu,W.L., Qiu,C.X., Wahlin,A., Winblad,B., & Fratiglioni,L. Diabetes mellitus and risk of dementia in the Kungsholmen project: a 6-year follow-up study. Neurology 63, 1181-1186 (2004).

4. Last,D. et al. Global and regional effects of type 2 diabetes mellitus on brain tissue volumes and cerebral vasoreactivity . Diabetes Care 30, 1193-1199 (2007).

5. Novak,V. et al. White matter hyperintensities and dynamics of postural control. Magnetic Resonance Imaging 27, 752-759 (2009).

6. Tiehuis,A.M. et al. Cerebral perfusion in relation to cognitive function and type 2 diabetes. Diabetologia 51, 1321-1326 (2008).

7. Aviles-Olmos I, Limousin P, Lees A, Foltynie T. Parkinson's disease, insulin resistance and novel agents of neuroprotection. Brain. 2013 136(Pt 2):374-84.

8. Santiago JA, Potashkin JA. Shared dysregulated pathways lead to Parkinson's disease and diabetes. rends Mol Med. 2013 19(3):176-86.

9. Born,J. et al. Sniffing neuropeptides: a transnasal approach to the human brain. Nat. Neurosci. 5, 514-516 (2002).

10. Reger,M.A. & Craft,S. Intranasal insulin administration: a method for dissociating central and peripheral effects of insulin. Drugs Today (Barc. ) 42, 729-739 (2006).

11. Derakhshan,F. & Toth,C. Insulin and the brain. Curr. Diabetes Rev 9, 102-116 (2013).

12. Hanson,L.R. & Frey,W.H. Intranasal delivery bypasses the blood-brain barrier to target therapeutic agents to the central nervous system and treat neurodegenerative disease. BMC. Neurosci. 9 Suppl 3, S5 (2008).

13. Gunning-Dixon,F.M. & Raz,N. The cognitive correlates of white matter abnormalities in normal aging: a quantitative review. Neuropsychology 14, 224-232 (2000).

14.Thorne,R.G., Pronk,G.J., Padmanabhan,V., & Frey,W.H.2. Delivery of insulin-like growth factor-I to the rat brain and spinal cord along olfactory and trigeminal pathways following intranasal administration. Neuroscience 127, 481-496 (2004).

15. Hallschmid,M. et al. Towards the therapeutic use of intranasal neuropeptide administration in metabolic and cognitive disorders. Regul. Pept. 149, 79-83 (2008).

16. Benedict,C. et al. Intranasal insulin improves memory in humans. Psychoneuroendocrinology 29, 1326-1334 (2004).

17. Strachan,M.W. Insulin and cognitive function in humans: experimental data and therapeutic considerations. Biochem. Soc. Trans. 33, 1037-1040 (2005).

18.Reger,M.A. et al. Effects of intranasal insulin on cognition in memory-impaired older adults: modulation by APOE genotype. Neurobiol. Aging 27, 451-458 (2006).

19. Benedict,C. et al. Intranasal insulin improves memory in humans: superiority of insulin aspart. Neuropsychopharmacology 32, 239-243 (2007).

20. Reger,M.A. et al. Intranasal insulin improves cognition and modulates beta-amyloid in early AD. Neurology 70, 440-448 (2008).

21. Benedict,C. et al. Intranasal insulin as a therapeutic option in the treatment of cognitive impairments. Exp. Gerontol. 46, 112-115 (2011).

22.Shemesh,E., Rudich,A., Harman-Boehm,I., & Cukierman-Yaffe,T. Effect of intranasal insulin on cognitive function: a systematic review. J Clin. Endocrinol. Metab 97, 366-376 (2012).

23.Novak,V. et al. Enhancement of Vasoreactivity and Cognition by Intranasal Insulin in Type 2 Diabetes. Diabetes Care(2013).

24.Biessels,G.J., Staekenborg,S., Brunner,E., Brayne,C., & Scheltens,P. Risk of dementia in diabetes mellitus: a systematic review. Lancet Neurol. 5, 64-74 (2006).

25.Reijmer,Y.D. et al. Accelerated cognitive decline in patients with type 2 diabetes: MRI correlates and risk factors. Diabetes Metab Res. Rev. 27, 195-202 (2011).

26. Korf,E.S., White,L.R., Scheltens,P., & Launer,L.J. Brain aging in very old men with type 2 diabetes: the Honolulu-Asia Aging Study. Diabetes Care 29, 2268-2274 (2006).

27. Novak,V. et al. Adhesion molecules, altered vasoreactivity, and brain atrophy in type 2 diabetes. Diabetes Care 34, 2438-2441 (2011).

28. Manor,B., Newton,E., Abduljalil,A., & Novak,V. The relationship between brain volume and walking outcomes in older adults with and without diabetic peripheral neuropathy. Diabetes Care 35, 1907-1912 (2012).

29. Cukierman-Yaffe,T. et al. Relationship between baseline glycemic control and cognitive function in individuals with type 2 diabetes and other cardiovascular risk factors: the action to control cardiovascular risk in diabetes-memory in diabetes (ACCORD-MIND) trial. Diabetes Care 32, 221-226 (2009).

30. Cukierman-Yaffe,T. et al. Relationship between baseline glycemic control and cognitive function in individuals with type 2 diabetes and other cardiovascular risk factors: the action to control cardiovascular risk in diabetes-memory in diabetes (ACCORD-MIND) trial. Diabetes Care 32, 221-226 (2009).

31. Launer,L.J. et al. Effects of intensive glucose lowering on brain structure and function in people with type 2 diabetes (ACCORD MIND): a randomised open-label substudy. Lancet Neurol. 10, 969-977 (2011).

32. Craft,S., Cholerton,B., & Baker,L.D. Insulin and Alzheimer's disease: untangling the web. J Alzheimers. Dis. 33 Suppl 1, S263-S275 (2013).

33. Morris,J.K. & Burns,J.M. Insulin: an emerging treatment for Alzheimer's disease dementia? Curr. Neurol. Neurosci. Rep. 12, 520-527 (2012).

34. Benedict,C., Hallschmid,M., Schultes,B., Born,J., & Kern,W. Intranasal insulin to improve memory function in humans. Neuroendocrinology 86, 136-142 (2007).

35. Benedict,C. et al. Intranasal insulin improves memory in humans: superiority of insulin aspart. Neuropsychopharmacology 32, 239-243 (2007).

36. Reger,M.A. et al. Intranasal Insulin Administration Dose-Dependently Modulates Verbal Memory and Plasma Amyloid-beta in Memory-Impaired Older Adults. J. Alzheimers. Dis. 13, 323-331 (2008).

37. Leary,A.C., Stote,R.M., Breedt,H.J., O'Brien,J., & Buckley,B. Pharmacokinetics and pharmacodynamics of intranasal insulin administered to healthy subjects in escalating doses. Diabetes Technol. Ther. 7, 124-130 (2005).

38. Freiherr,J. et al. Intranasal Insulin as a Treatment for Alzheimer's Disease: A Review of Basic Research and Clinical Evidence. CNS. Drugs(2013).

39. Messier,C. & Teutenberg,K. The role of insulin, insulin growth factor, and insulin-degrading enzyme in brain aging and Alzheimer's disease. Neural Plast. 12, 311-328 (2005).

40. Watson,G.S. et al. Insulin effects on CSF norepinephrine and cognition in Alzheimer's disease. Neurobiol. Aging 27, 38-41 (2006).

41. Schilling,T.M. et al. Intranasal insulin increases regional cerebral blood flow in the insular cortex in men independently of cortisol manipulation. Hum. Brain Mapp.(2013).

42. Launer,L.J. Diabetes and brain aging: epidemiologic evidence. Curr. Diab. Rep. 5, 59-63 (2006).

43. Plum,L., Schubert,M., & Bruning,J.C. The role of insulin receptor signaling in the brain. Trends Endocrinol. Metab 16, 59-65 (2005).

44. Plum,L., Belgardt,B.F., & Bruning,J.C. Central insulin action in energy and glucose homeostasis. J Clin. Invest 116, 1761-1766 (2006).

45. Figlewicz,D.P. Insulin, food intake, and reward. Semin. Clin. Neuropsychiatry 8, 82-93 (2003).

46. Figlewicz,D.P. & Benoit,S.C. Insulin, leptin, and food reward: update 2008. Am. J Physiol Regul. Integr. Comp Physiol 296, R9-R19 (2009).

47. Stice,E., Figlewicz,D.P., Gosnell,B.A., Levine,A.S., & Pratt,W.E. The contribution of brain reward circuits to the obesity epidemic. Neurosci. Biobehav. Rev(2012).

48. Hopkins,D.F. & Williams,G. Insulin receptors are widely distributed in human brain and bind human and porcine insulin with equal affinity. Diabet. Med. 14, 1044-1050 (1997).

49. Albrecht,J., Wroblewska,B., & Mossakowski,M.J. The binding of insulin to cerebral capillaries and astrocytes of the rat. Neurochemical Research 7, 489-494 (1981).

50. Banks,W.A. The source of cerebral insulin. Eur. J. Pharmacol. 490, 5-12 (2004).

51. Horsch,D. & Kahn,C.R. Region-specific mRNA expression of phosphatidylinositol 3-kinase regulatory isoforms in the central nervous system of C57BL/6J mice. J Comp Neurol. 415, 105-120 (1999).

52. Cersosimo,E. & DeFronzo,R.A. Insulin resistance and endothelial dysfunction: the road map to cardiovascular diseases. Diabetes Metab Res Rev 22, 423-436 (2006).

53. Girouard,H. & Iadecola,C. Neurovascular coupling in the normal brain and in hypertension, stroke, and Alzheimer disease. J. Appl. Physiol 100, 328-335 (2006).

54. Abbott,M.A., Wells,D.G., & Fallo,J.R. The Insulin Receptor Tyrosine Kinase Substrate p58/53 and the Insulin Receptor Are Components of CNS Synapses. Journal of Neuroscience 19, 7300-7308 (1999).

55. Lok,J. et al. Cell–cell Signaling in the Neurovascular Unit. Neurochemical Research 32, 0364-3190 (2007).

56. Amir,S. & Shechter,Y. Centrally mediated hypoglycemic effect of insulin: apparent involvement of specific insulin receptors. Brain Res. 418, 152-156 (1987).

57. Cranston,I. et al. Regional differences in cerebral blood flow and glucose utilization in diabetic man: the effect of insulin. J Cereb Blood Flow Metab 18, 130-140 (1998).

58. Kim,J.A., Montagnani,M., Koh,K.K., & Quon,M.J. Reciprocal relationships between insulin resistance and endothelial dysfunction: molecular and pathophysiological mechanisms. Circulation 113, 1888-1904 (2006).

59. Muniyappa,R., Montagnani,M., Koh,K.K., & Quon,M.J. Cardiovascular actions of insulin. Endocr. Rev. 28, 463-491 (2007).

60. Hanson,L.R. & Frey,W.H. Intranasal delivery bypasses the blood-brain barrier to target therapeutic agents to the central nervous system and treat neurodegenerative disease. BMC. Neurosci. 9 Suppl 3, S5 (2008).

61. Farris,W. et al. Insulin-degrading enzyme regulates the levels of insulin, amyloid beta-protein, and the beta-amyloid precursor protein intracellular domain in vivo. Proc. Natl. Acad. Sci U. S. A 100, 4162-4167 (2003).

62. Carro,E., Trejo,J.L., Nunez,A., & Torres-Aleman,I. Brain repair and neuroprotection by serum insulin-like growth factor I. Mol. Neurobiol. 27, 153-162 (2003).

63. Carro,E. & Torres-Aleman,I. Serum insulin-like growth factor I in brain function. Keio J Med 55, 59-63 (2006).

64. Francis,G.J. et al. Intranasal insulin prevents cognitive decline, cerebral atrophy and white matter changes in murine type I diabetic encephalopathy. Brain 131, 3311-3334 (2008).

65. Margolis,R.U. & Altszuler,N. Insulin in the cerebrospinal fluid. Nature 215, 1375-1376 (1967).

66. King,G.L. & Johnson,S.M. Receptor-mediated transport of insulin across endothelial cells. Science 227, 1583-1586 (1985).

67. Banks,W.A. Brain meets body: the blood-brain barrier as an endocrine interface. Endocrinology 153, 4111-4119 (2012).

68. Banks,W.A. Blood-brain barrier as a regulatory interface. Forum Nutr. 63, 102-110 (2010).

69. Hallschmid,M., Benedict,C., Born,J., & Kern,W. Targeting metabolic and cognitive pathways of the CNS by intranasal insulin administration. Expert. Opin. Drug Deliv. 4, 319-322 (2007).

70. Makimattila,S. & Yki-Jarvinen,H. Endothelial dysfunction in human diabetes. Curr Diab Rep 2, 26-36 (2002).

71. Brownlee,M. The pathobiology of diabetic complications. Diabetes 54, 1615-1625 (2006).

72. Kilpatrick,E.S., Rigby,A.S., & Atkin,S.L. For debate. Glucose variability and diabetes complication risk: we need to know the answer. Diabet. Med. 27, 868-871 (2010).

73. Manschot,S.M. et al. Brain magnetic resonance imaging correlates of impaired cognition in patients with type 2 diabetes. Diabetes 55, 1106-1113 (2006).

74. Manschot,S.M. et al. Metabolic and vascular determinants of impaired cognitive performance and abnormalities on brain magnetic resonance imaging in patients with type 2 diabetes. Diabetologia 50, 2388-2397 (2007).

75. Thorne,R.G., Lakkaraju,A., Rodriguez-Boulan,E., & Nicholson,C. In vivo diffusion of lactoferrin in brain extracellular space is regulated by interactions with heparan sulfate. Proc. Natl. Acad. Sci. U. S. A 105, 8416-8421 (2008).

76. Thorne,R.G., Hanson,L.R., Ross,T.M., Tung,D., & Frey,W.H.2. Delivery of interferon-beta to the monkey nervous system following intranasal administration. Neuroscience 152, 785-797 (2008).

77. Thorne,R.G. & Frey,W.H.2. Delivery of neurotrophic factors to the central nervous system: pharmacokinetic considerations. Clin. Pharmacokinet. 40, 907-946 (2001).

78. Balin,B.J., Boradwell,R.D., Salcman,M., & el-Kalliny,M. Avenues for entry of peripherally administered protein to the central nervous system in mouse, rat and squirrel monkey. J. Comp. Neurol. 251, 260-280 (1986).

79. Moses,A.C., Gordon,G.S., Carey,M.C., & Flier,J.S. Insulin administered intranasally as an insulin-bile salt aerosol. Effectiveness and reproducibility in normal and diabetic subjects. Diabetes 32, 1040-1047 (1983).

80. Leary,A.C., Dowling,M., Cussen,K., O'Brien,J., & Stote,R.M. Pharmacokinetics and pharmacodynamics of intranasal insulin spray (Nasulin) administered to healthy male volunteers: infuence of the nasal cycle. J Diabetes Sci Technol. 2, 1054-1060 (2008).

81. Stote,R., Miller,M., Marbury,T., Shi,L., & Strange,P. Enhanced absorption of Nasulin, an ultrarapid-acting intranasal insulin formulation, using single nostril administration in normal subjects. J Diabetes Sci Technol. 5, 113-119 (2011).

82. Stote,R., Marbury,T., Shi,L., Miller,M., & Strange,P. Comparison pharmacokinetics of two concentrations (0.7% and 1.0%) of Nasulin, an ultra-rapid-acting intranasal insulin formulation. J Diabetes Sci Technol. 4, 603-609 (2010).

83. Coates,P.A. et al. Intranasal insulin: the effects of three dose regimens on postprandial glycaemic profiles in type II diabetic subjects. Diabet. Med. 12, 235-239 (1995).

84. Frauman,A.G., Cooper,M.E., Parsons,B.J., Jerums,G., & Louis,W.J. Long-term use of intranasal insulin in insulin-dependent diabetic patients. Diabetes Care 10, 573-578 (1987).

85. Frauman,A.G., Jerums,G., & Louis,W.J. Effects of intranasal insulin in non-obese type II diabetics. Diabetes Res. Clin. Pract. 3, 197-202 (1987).

86. Lalej-Bennis,D. et al. Efficacy and tolerance of intranasal insulin administered during 4 months in severely hyperglycaemic Type 2 diabetic patients with oral drug failure: a cross-over study. Diabet. Med 18, 614-618 (2001).

87. Frauman,A.G., Jerums,G., & Louis,W.J. Effects of intranasal insulin in non-obese type II diabetics. Diabetes Res Clin. Pract. 3, 197-202 (1987).

88. Lalej-Bennis,D. et al. Efficacy and tolerance of intranasal insulin administered during 4 months in severely hyperglycaemic Type 2 diabetic patients with oral drug failure: a cross-over study. Diabet. Med. 18, 614-618 (2001).

89. Heni,M. et al. Nasal insulin changes peripheral insulin sensitivity simultaneously with altered activity in homeostatic and reward-related human brain regions. Diabetologia 55, 1773-1782 (2012).

90. Benedict,C. et al. Immediate but not long-term intranasal administration of insulin raises blood pressure in human beings. Metabolism 54, 1356-1361 (2005).

91. Benedict,C., Kern,W., Schultes,B., Born,J., & Hallschmid,M. Differential sensitivity of men and women to anorexigenic and memory-improving effects of intranasal insulin. J. Clin. Endocrinol. Metab 93, 1339-1344 (2008).

92. McInnes,G.T. The expanding role of angiotensin receptor blockers in the management of the elderly hypertensive. Curr Med. Res. Opin. 19, 452-455 (2003).

93. Craft,S. Insulin resistance and cognitive impairment: a view through the prism of epidemiology. Arch. Neurol. 62, 1043-1044 (2005).

94. Craft,S. Insulin resistance syndrome and Alzheimer's disease: age- and obesity-related effects on memory, amyloid, and inflammation. Neurobiol. Aging 26 Suppl 1, 65-69 (2005).

95. Cedernaes,J. et al. Intranasal insulin targeting to brain and cerebrospinal fluid (CSF): A review of its clinical effects and mechanisms. Respiratory Drug Delivery60-67 (2013).

96. Banks,W.A., Owen,J.B., & Erickson,M.A. Insulin in the brain: there and back again. Pharmacol. Ther. 136, 82-93 (2012).

97. Cholerton,B., Baker,L.D., & Craft,S. Insulin, cognition, and dementia. Eur. J Pharmacol.(2013).

98. Cholerton,B. et al. Insulin and sex interactions in older adults with mild cognitive impairment. J Alzheimers. Dis. 31, 401-410 (2012).

99. Park,C.R., Seeley,R.J., Craft,S., & Woods,S.C. Intracerebroventricular insulin enhances memory in a passive-avoidance task. Physiol Behav. 68, 509-514 (2000).

100. Reger,M.A. et al. Intranasal insulin administration dose-dependently modulates verbal memory and plasma amyloid-beta in memory-impaired older adults. J Alzheimers. Dis. 13, 323-331 (2008).

101. Schioth,H.B., Craft,S., Brooks,S.J., Frey,W.H., & Benedict,C. Brain insulin signaling and Alzheimer's disease: current evidence and future directions. Mol. Neurobiol. 46, 4-10 (2012).

102. Watson,G.S. et al. Insulin increases CSF Abeta42 levels in normal older adults. Neurology 60, 1899-1903 (2003).

103. Benedict,C., Hallschmid,M., Schultes,B., Born,J., & Kern,W. Intranasal insulin to improve memory function in humans. Neuroendocrinology 86, 136-142 (2007).

104. Reger,M.A. et al. Intranasal insulin administration dose-dependently modulates verbal memory and plasma amyloid-beta in memory-impaired older adults. J. Alzheimers. Dis. 13, 323-331 (2008).

105. Craft,S. & Watson,G.S. Insulin and neurodegenerative disease: shared and specific mechanisms. Lancet Neurol. 3, 169-178 (2004).

106. de la Monte,S.M. Contributions of brain insulin resistance and deficiency in amyloid-related neurodegeneration in Alzheimer's disease. Drugs 72, 49-66 (2012).

107. Chapman,C.D. et al. Intranasal treatment of central nervous system dysfunction in humans. Pharm. Res 30, 2475-2484 (2013).

108. Dufor,O., Serniclaes,W., Sprenger-Charolles,L., & Demonet,J.F. Top-down processes during auditory phoneme categorization in dyslexia: a PET study. Neuroimage. 34, 1692-1707 (2007).

109. Abboud,H., Berroir,S., Labreuche,J., Orjuela,K., & Amarenco,P. Insular involvement in brain infarction increases risk for cardiac arrhythmia and death. Ann. Neurol. 59, 691-699 (2006).

110. Jauch-Chara,K. et al. Intranasal insulin suppresses food intake via enhancement of brain energy levels in humans. Diabetes 61, 2261-2268 (2012).

111. Kullmann,S. et al. Intranasal insulin modulates intrinsic reward and prefrontal circuitry of the human brain in lean women. Neuroendocrinology 97, 176-182 (2013).

112. Zhao,P. et al. Altered vasoreactivity and peri-infarct hyperintensities affect multiple terriories in stroke. Neurology 72, 643-649 (2009).

113. Eckert,M.A. et al. At the heart of the ventral attention system: the right anterior insula. Hum. Brain Mapp. 30, 2530-2541 (2009).

114. Figlewicz,D.P. et al. Neurotransmitter transporters: target for endocrine regulation. Horm. Metab Res 31, 335-339 (1999).

115. Benedict,C., Kern,W., Schultes,B., Born,J., & Hallschmid,M. Differential sensitivity of men and women to anorexigenic and memory-improving effects of intranasal insulin. J Clin. Endocrinol. Metab 93, 1339-1344 (2008).

116. Hallschmid,M. et al. Intranasal insulin reduces body fat in men but not in women. Diabetes 53, 3024-3029 (2004).

117. Hallschmid,M., Benedict,C., Schultes,B., Born,J., & Kern,W. Obese men respond to cognitive but not to catabolic brain insulin signaling. Int. J Obes. (Lond) 32, 275-282 (2008).

118. Hallschmid,M., Benedict,C., Schultes,B., Born,J., & Kern,W. Obese men respond to cognitive but not to catabolic brain insulin signaling. Int. J. Obes. (Lond) 32, 275-282 (2008).

119. Yang,Y. et al. Intranasal insulin ameliorates tau hyperphosphorylation in a rat model of type 2 diabetes. J Alzheimers. Dis. 33, 329-338 (2013).

120. Krug,R., Benedict,C., Born,J., & Hallschmid,M. Comparable sensitivity of postmenopausal and young women to the effects of intranasal insulin on food intake and working memory. J Clin. Endocrinol. Metab 95, E468-E472 (2010).

121. Chistyakova,O.V., Bondareva,V.M., Shipilov,V.N., Sukhov,I.B., & Shpakov,A.O. Intranasal administration of insulin eliminates the deficit of long-term spatial memory in rats with neonatal diabetes mellitus. Dokl. Biochem. Biophys. 440, 216-218 (2011).

122. Alsop,D.C. & Press,D.Z. Activation and baseline changes in functional MRI studies of Alzheimer disease. Neurology 69, 1645-1646 (2007).

123. Dai,W., Garcia,D., de,B.C., & Alsop,D.C. Continuous flow-driven inversion for arterial spin labeling using pulsed radio frequency and gradient fields. Magn Reson. Med 60, 1488-1497 (2008).

124. Novak,V. et al. Cerebral blood flow velocity and periventricular white matter hyperintensities in type 2 diabetes. Diabetes Care 29, 1529-1534 (2006).

125. Cui,X., Abduljalil,A., Manor,B., Peng,C.K., & Novak,V. Multi-Scale glycemic variability: A link to gray matter atrophy and cognitive decline in type 2 diabetes. PLoS. One.(2013).

126. Franke,K., Gaser,Ch., Manor,B., & Novak,V. Advanced BrainAge in older adults with type 2 diabetes mellitus. Frontiers in Aging Neuroscience(2013).

127. Selim,M., Jones,R., Novak,P., Zhao,P., & Novak,V. The effects of body mass index on cerebral blood flow velocity. Clin. Auton. Res. 18, 331-338 (2008).

128. Hajjar,I., Selim,M., Novak,P., & Novak,V. The relationship between nighttime dipping in blood pressure and cerebral hemodynamics in nonstroke patients. J. Clin. Hypertens. (Greenwich. ) 9, 929-936 (2007).

129. Hajjar,I. et al. Association of Blood Pressure Elevation and Nocturnal Dipping With Brain Atrophy, Perfusion and Functional Measures in Stroke and Nonstroke Individuals. Am. J. Hypertens.(2009).

130. Hu,K., Peng,C.K., Czosnyka,M., Zhao,P., & Novak,V. Nonlinear assessment of cerebral autoregulation from spontaneous blood pressure and cerebral blood flow fluctuations. Cardiovasc. Eng 8, 60-71 (2008).

131. Manor,B. & Li,L. Characteristics of functional gait among people with and without peripheral neuropathy. Gait. Posture. 30, 253-256 (2009).

132. Sorond,F.A., Schnyer,D.M., Serrador,J.M., Milberg,W.P., & Lipsitz,L.A. Cerebral blood flow regulation during cognitive tasks: effects of healthy aging. Cortex 44, 179-184 (2008).

133. Sorond,F.A. et al. Neurovascular coupling is impaired in slow walkers: the MOBILIZE Boston Study. Ann. Neurol. 70, 213-220 (2011).

134. Albert,M.S., Heller,H.S., & Milberg,W. Changes in naming ability with age. Psychol. Aging 3, 173-178 (1988).

135. Munshi,M. et al. Cognitive dysfunction is associated with poor diabetes control in older adults. Diabetes Care 29, 1794-1799 (2006).

136. Kuo,H.K., Leveille,S.G., Yu,Y.H., & Milberg,W.P. Cognitive function, habitual gait speed, and late-life disability in the National Health and Nutrition Examination Survey (NHANES) 1999-2002. Gerontology 53, 102-110 (2007).

137. Kuo,H.K. et al. Cognitive function in normal-weight, overweight, and obese older adults: an analysis of the Advanced Cognitive Training for Independent and Vital Elderly cohort. J Am. Geriatr. Soc. 54, 97-103 (2006).

138. Zade,D. et al. Apolipoprotein epsilon 4 allele modifies waist-to-hip ratio effects on cognition and brain structure. J Stroke Cerebrovasc. Dis. 22, 119-125 (2013).

139. Levine,B., Stuss,D.T., & Milberg,W.P. Effects of aging on conditional associative learning: process analyses and comparison with focal frontal lesions. Neuropsychology. 11, 367-381 (1997).

140. Rudolph,J.L. et al. Impaired executive function is associated with delirium after coronary artery bypass graft surgery. J Am. Geriatr. Soc. 54, 937-941 (2006).

141. Leritz,E.C., McGlinchey,R.E., Kellison,I., Rudolph,J.L., & Milberg,W.P. Cardiovascular Disease Risk Factors and Cognition in the Elderly. Curr. Cardiovasc. Risk Rep. 5, 407-412 (2011).

142. Kuo,H.K. et al. Effect of blood pressure on cognitive functions in elderly persons. J Gerontol. A Biol. Sci Med Sci 59, 1191-1194 (2004).

143. Morra,L., Zade,D., McGlinchey,R.E., & Milberg,W.P. Normal aging and cognition: the unacknowledged contribution of cerebrovascular risk factors. Neuropsychol. Dev. Cogn B Aging Neuropsychol. Cogn 20, 271-297 (2013).

144. Hajjar,I. et al. A novel aging phenotype of slow gait, impaired executive function, and depressive symptoms: relationship to blood pressure and other cardiovascular risks. J. Gerontol. A Biol. Sci. Med. Sci. 64, 994-1001 (2009).

145. Kuo,H.K. et al. Effect of blood pressure and diabetes mellitus on cognitive and physical functions in older adults: a longitudinal analysis of the advanced cognitive training for independent and vital elderly cohort. J. Am. Geriatr. Soc. 53, 1154-1161 (2005).

146. Munshi,M.N. et al. Assessment of barriers to improve diabetes management in older adults: a randomized controlled study. Diabetes Care 36, 543-549 (2013).

147. Munshi,M.N. et al. Frequent hypoglycemia among elderly patients with poor glycemic control. Arch. Intern. Med. 171, 362-364 (2011).

148. Munshi,M.N., Maguchi,M., & Segal,A.R. Treatment of type 2 diabetes in the elderly. Curr. Diab. Rep. 12, 239-245 (2012).

149. Rejeski,W.J. et al. Correlates of health-related quality of life in overweight and obese adults with type 2 diabetes. Obesity. (Silver. Spring) 14, 870-883 (2006).

150. Rejeski,W.J., Ip,E.H., Katula,J.A., & White,L. Older adults' desire for physical competence. Med Sci Sports Exerc. 38, 100-105 (2006).

151. Rejeski,W.J. & Brawley,L.R. Functional health: innovations in research on physical activity with older adults. Med Sci Sports Exerc. 38, 93-99 (2006).

152. Wing,R.R. et al. Cardiovascular effects of intensive lifestyle intervention in type 2 diabetes. N. Engl. J Med 369, 145-154 (2013).

153. Foley,J.M. et al. Interactive effects of ApoE4 and diabetes risk ob latermyelinating white matter regions in neurologically healthy older aged adults. American Journal of Alzheimer'sDisease and Related Dementias In Press, (2013).

154. Harris,P.A. et al. Research electronic data capture (REDCap)--a metadata-driven methodology and workflow process for providing translational research informatics support. J Biomed. Inform. 42, 377-381 (2009).

155. Borlawsky,T.B., Lele,O., Jensen,D., Hood,N.E., & Wewers,M.E. Enabling distributed electronic research data collection for a rural Appalachian tobacco cessation study. J Am. Med Inform. Assoc. 18 Suppl 1, i140-i143 (2011).

156. Franklin,J.D., Guidry,A., & Brinkley,J.F. A partnership approach for Electronic Data Capture in small-scale clinical trials. J Biomed. Inform. 44 Suppl 1, S103-S108 (2011).

157. Moon,H.S. & Mantzoros,C.S. Adiponectin and metformin additively attenuate IL1beta-induced malignant potential of colon cancer. Endocr. Relat Cancer 20, 849-859 (2013).

158. Hee,P.K. et al. Circulating irisin in relation to insulin resistance and the metabolic syndrome. J Clin. Endocrinol. Metab 98, 4899-4907 (2013).

159. Dalamaga,M. et al. Leptin at the intersection of neuroendocrinology and metabolism: current evidence and therapeutic perspectives. Cell Metab 18, 29-42 (2013).

160. Huffman,D.M. et al. Abdominal obesity, independent from caloric intake, accounts for the development of intestinal tumors in Apc(1638N/+) female mice. Cancer Prev. Res (Phila) 6, 177-187 (2013).

161. Kizer,J.R. et al. Total and high-molecular-weight adiponectin and risk of coronary heart disease and ischemic stroke in older adults. J Clin. Endocrinol. Metab 98, 255-263 (2013).

162. Berthold,H.K., Berneis,K., Mantzoros,C.S., Krone,W., & Gouni-Berthold,I. Effects of simvastatin and ezetimibe on interleukin-6 and high-sensitivity C-reactive protein. Scand. Cardiovasc. J Suppl 47, 20-27 (2013).

163. Moon,H.S. et al. Salutary effects of adiponectin on colon cancer: in vivo and in vitro studies in mice. Gut 62, 561-570 (2013).

164. Wilkinson,D., Ko,P., Kilduff,P., McGlinchey,R., & Milberg,W. Improvement of a face perception deficit via subsensory galvanic vestibular stimulation. J Int. Neuropsychol. Soc. 11, 925-929 (2005).

165. Barrett,A.M. et al. Cognitive rehabilitation interventions for neglect and related disorders: moving from bench to bedside in stroke patients. J Cogn Neurosci. 18, 1223-1236 (2006).

166. Eggermont,L.H., Milberg,W.P., Lipsitz,L.A., Scherder,E.J., & Leveille,S.G. Physical activity and executive function in aging: the MOBILIZE Boston Study. J Am. Geriatr. Soc. 57, 1750-1756 (2009).

167. Lindemer,E.R., Salat,D.H., Leritz,E.C., McGlinchey,R.E., & Milberg,W.P. Reduced cortical thickness with increased lifetime burden of PTSD in OEF/OIF Veterans and the impact of comorbid TBI. Neuroimage. Clin. 2, 601-611 (2013).

168. Stricker,N.H. et al. Decreased white matter integrity in neuropsychologically defined mild cognitive impairment is independent of cortical thinning. J Int. Neuropsychol. Soc. 19, 925-937 (2013).

169. Alsop,D.C. Improved efficiency for multi-slice continuous arterial spin labeling using time-varying gradients. Proceedings of the International Society for Magnetic Resonance in medicine, 9th Scientific meeting and exhibition. International Society for Magnetic resonance in Medicine., 1561. 2000.

Ref Type: Abstract

170. Alsop,D.C. & Detre,J.A. Multisection cerebral blood flow MR imaging with continuous arterial spin labeling. Radiology 208, 410-416 (1998).

171. Alsop,D.C., Detre,J.A., & Grossman,M. Assessment of cerebral blood flow in Alzheimer's disease by spin-labeled magnetic resonance imaging. Ann. Neurol. 47, 93-100 (2000).

172. Alsop,D.C., Casement,M., Fong,T., & Press,D.Z. Perfusion MRI reveals elevated function of temporal and inferior frontal regions in early AD. Neurobiol Aging 25, S296-297 (2004).

173. Williams,D.S., Detre,J.A., Leigh,J.S., & Koretsky,A.P. Magnetic Resonance Imaging of Perfusion Using Spin Inversion of Arterial Water. Proceedings of the National Academy of Sciences USA 89, 212-216 (1992).

174. Reger,M.A. & Craft,S. Intranasal insulin administration: a method for dissociating central and peripheral effects of insulin. Drugs Today (Barc. ) 42, 729-739 (2006).

175. Arauz-Pacheco,C., Parrott,M.A., & Raskin,P. Hypertension management in adults with diabetes. Diabetes Care 27 Suppl 1, S65-S67 (2004).

176. American Diabetes Association Standards of medical care in diabetes--2012. Diabetes Care 35 Suppl 1, S11-S63 (2012).

177. Pani,L.N. et al. Effect of aging on A1C levels in individuals without diabetes: evidence from the Framingham Offspring Study and the National Health and Nutrition Examination Survey 2001-2004. Diabetes Care 31, 1991-1996 (2008).

178. Benedict,R.H.B., Schretlen,D., Groninger,L., Dobraski,M., & Sphritz,B. Revision of the Brief VisuospatilaaMemory test: Studies of normal perfromance, reliability and validity. Psycholoigcal Assessment 8, 145-153 (1996).

179. Yeudall,L.T., Fromm,D., Reddon,J.R., & Stefanyk,W.O. Normative data stratified by age and sex for 12 neuropsychological tests. Journal of Clinical Psychology 42, 918-946 (1986).

180. Shapiro,A., Benedict,R., Schretlen,D., & Brandt,J. Construct and concurrent validity of the Hopkins Verbal Learning Test--Revised. Clinical Neuropsychologist 13, 348-358 (1999).

181. Folstein,M.F., Folstein,S.E., & McHugh,P.R. Mini-mental state. A practical method for grading the cognitive state of patients for the clinician. J Psychiatr Res 12, 189-198 (1975).

182. Goldschmidt,T.J., Mallin,R., & Still,C.N. Recognition of cognitive impairment in primary care outpatients. South Med J 76, 1264-1270 (1983).

183. Murden,R.A., McRae,T.D., Kaner,S., & Bucknam,M.E. Mini-Mental State exam scores vary with education in blacks and whites. J Am Geriatr Soc 39, 149-155 (1991).

184. Tangalos,E.G. et al. The Mini-Mental State Examination in general medical practice: clinical utility and acceptance. Mayo Clin Proc 71, 829-837 (1996).

185. George,L., Landerman,R., Blazer,D., & Anthony,J.in Cognitive Impairment, in Psychiatric Disorders in America (eds. Robins,L. & Regier,D.) 291-327 (The Free Press, New York, 1991).

186. Saczynski,J.S. et al. Cognitive impairment: an increasingly important complication of type 2 diabetes: the age, gene/environment susceptibility--Reykjavik study. Am. J Epidemiol. 168, 1132-1139 (2008).

187. Saczynski,J.S., McManus,D.D., & Goldberg,R.J. Commonly Used Data-collection Approaches in Clinical Research. Am. J Med(2013).

188. Lasselin,J. et al. Fatigue and cognitive symptoms in patients with diabetes: relationship with disease phenotype and insulin treatment. Psychoneuroendocrinology 37, 1468-1478 (2012).

189. Owen,A.M., Downes,J.J., Sahakian,B.J., Polkey,C.E., & Robbins,T.W. Planning and spatial working memory following frontal lobe lesions in man. Neuropsychologia 28, 1021-1034 (1990).

190. Kaufmann,L. et al. Neurocognition and brain structure in pediatric patients with type 1 diabetes. J. of Pediatric Neuroradiology25-35 (2012).

191. Saczynski,J.S. et al. Glycemic status and brain injury in older individuals: the age gene/environment susceptibility-Reykjavik study. Diabetes Care 9, 1608-1613 (2009).

192.Alsop,D.C. & Detre,J.A. Reduced transit-time sensitivity in non-invasive magnetic resonance imaging of human cerebral blood flow. Journal Cerebral Blood Flow Metabolism 16, 1236-1249 (1996).

193.Buxton,R.B., Wong,E.C., & Frank,L.R. Dynamics of blood flow and oxygenation changes during brain activation: the balloon model. Magn Reson Med 39, 855-864 (1998).

194.Buxton,R.B. Quantifying CBF with arterial spin labeling. J Magn Reson Imaging 22, 723-726 (2005).

195.Wang,J. et al. Comparison of quantitative perfusion imaging using arterial spin labeling at 1.5 and 4.0 Tesla. Magn Reson Med 48, 242-254 (2002).

196.Herscovitch,P. & Raichle,M.E. What is the correct value for the brain--blood partition coefficient for water? J Cereb. Blood Flow Metab 5, 65-69 (1985).

197.Lu,H., Clingman,C., Golay,X., & van Zijl,P.C.M. Determining the longitudinal relaxation time (T1) of blood at 3 Tesla. Magn Reson Med 52, 679-682 (2004).

198.Wallace,T.M., Levy,J.C., & Matthews,D.R. Use and abuse of HOMA modeling. Diabetes Care 27, 1487-1495 (2004).

199.Laird,N.M. & Ware,J.H. Random-Effects Models for Longitudinal Data. Biometrics 38, 963-974 (1982).

200.Paul,L., Ellis,B.M., Leese,G.P., McFadyen,A.K., & McMurray,B. The effect of a cognitive or motor task on gait parameters of diabetic patients, with and without neuropathy. Diabet. Med 26, 234-239 (2009).

201.Jones,B.L. & Nagin,D.S. Advances in group-based trajectory modeling and a SAS procedure for estimating them. Sociol.Methods res. 35, 542-571. 2007.

202.Studenski, S., S. Perera, K. Patel, C. Rosano, K. Faulkner, M. Inzitari, J. Brach, J. Chandler, P. Cawthon, E. B. Connor, M. Nevitt, M. Visser, S. Kritchevsky, S. Badinelli, T. Harris, A. B. Newman, J. Cauley, L. Ferrucci, and J. Guralnik. Gait speed and survival in older adults. JAMA 2011, 305:50-58

203.Novolin R, insert. http://www.novonordiskmedicalinformation.com//file_upload/Novolin%20R%20Prescribing%20Information,%20March%202013.pdf
